# BET inhibition curbs macrophage inflammation, lipid accumulation, and atherogenesis by disrupting the YAP/TAZ-BRD4 axis

**DOI:** 10.1093/jleuko/qiag017

**Published:** 2026-04-29

**Authors:** Praveen Krishna Veerasubramanian, Vijaykumar S Meli, Hamza Atcha, Wenqi Wang, Timothy L Downing, Wendy F Liu

**Affiliations:** Department of Biomedical Engineering, University of California Irvine, Irvine, CA 92697, United States; UCI Edwards Lifesciences Foundation Cardiovascular Innovation and Research Center (CIRC), University of California Irvine, Irvine, CA 92697, United States; Department of Biomedical Engineering, University of California Irvine, Irvine, CA 92697, United States; UCI Edwards Lifesciences Foundation Cardiovascular Innovation and Research Center (CIRC), University of California Irvine, Irvine, CA 92697, United States; Department of Biomedical Engineering, University of California Irvine, Irvine, CA 92697, United States; UCI Edwards Lifesciences Foundation Cardiovascular Innovation and Research Center (CIRC), University of California Irvine, Irvine, CA 92697, United States; Department of Developmental and Cell Biology, University of California Irvine, Irvine, CA 92697, United States; Department of Biomedical Engineering, University of California Irvine, Irvine, CA 92697, United States; UCI Edwards Lifesciences Foundation Cardiovascular Innovation and Research Center (CIRC), University of California Irvine, Irvine, CA 92697, United States; NSF-Simons Center for Multiscale Cell Fate Research, University of California Irvine, Irvine, CA 92697, United States; Department of Microbiology and Molecular Genetics, University of California Irvine, Irvine, CA 92697, United States; Department of Biomedical Engineering, University of California Irvine, Irvine, CA 92697, United States; UCI Edwards Lifesciences Foundation Cardiovascular Innovation and Research Center (CIRC), University of California Irvine, Irvine, CA 92697, United States; Chemical and Biomolecular Engineering, University of California Irvine, Irvine, CA 92697, United States; Department of Molecular Biology and Biochemistry, University of California Irvine, Irvine, CA 92697, United States; Institute for Immunology, University of California Irvine, Irvine, CA 92697, United States

**Keywords:** BRD4, inflammation, lipid, macrophage, YAP

## Abstract

Macrophage dysfunction is hallmark of atherosclerotic disease, characterized by inflammation and uptake of oxidized low-density lipoproteins. We investigate the role of the epigenetic reader bromodomain-containing protein 4 (BRD4) in orchestrating macrophage responses through interactions with the mechanosensitive transcriptional coactivators YAP/TAZ. Suppression of BRD4 via bromodomain and extra-terminal motif (BET) protein inhibitors (BETi) unveils a remarkable capacity to mitigate YAP/TAZ-driven inflammation. Knockdown of YAP, TAZ, or BRD4 in macrophages shows a significant convergence of inflammatory genes under the regulatory purview of these transcriptional regulators. In addition, persistent activation of YAP and TAZ initiates a partial inflammatory phenotype in macrophages, which is effectively ameliorated with BETi. Notably, CD36 and low-density lipoprotein receptor-1 (LOX1), pivotal receptors involved in uptake of oxidized low-density lipoprotein, emerge as direct YAP/TAZ targets. We employed a BD2-specific BETi, ABBV-744, in an AAV-PCSK9-induced atherosclerosis model to test the therapeutic potential of BET inhibition. Although reduction in cholesterol levels is modest, BETi substantially curtails plaque formation, diminishing macrophage infiltration, and suppressing the upregulation of YAP/TAZ and oxidized low-density lipoprotein uptake receptors associated with atherogenesis. Intriguingly, even in conditions marked by heightened YAP/TAZ expression induced by myeloid cell-targeted YAP/TAZ overexpression, BETi effectively dampens inflammation, mitigates foam cell formation, and disease progression. Our work underscores the considerable promise of targeting the YAP/TAZ-BRD4 axis as a therapeutic strategy for averting atherosclerosis, thereby disrupting the relentless cycle of inflammation, mechanosensory responses, and oxLDL uptake characteristic of atherosclerosis progression.

## Teaser

YAP/TAZ-BRD4 regulates macrophage inflammation and lipid accumulation

## 1. Introduction

Macrophage recruitment to the vessel wall and ingestion of modified lipoproteins such as oxidized low-density lipoprotein (oxLDL) is a key event in the progression of atherosclerosis.^1^ Consequently, these cells transform into foam cells that secrete additional inflammatory mediators.^2^ This pro-inflammatory macrophage polarization leads to elevated expression of inflammatory cytokines (such as tumor necrosis factor-α (TNFα), interleukin-6, and interleukin-1β), damaging free radicals, proteolytic enzymes, and chemokines that extend macrophage retention within the tissue.^1^ Upregulation of scavenger receptors such as CD36 and LOX1 potentiate the uptake of lipids and formation of foam cells.^2^ Over time, foam cells contribute to plaque formation, extracellular matrix remodeling, and vessel stiffening. Moreover, foam cells undergo necrotic or apoptotic death, further fueling inflammation and foam cell-mediated calcification.^1^ In this dynamic microenvironment, macrophages encounter diverse biomechanical and biochemical cues.^3^ However, targeting macrophage inflammation and lipid uptake by altering pathways involved in mechanosensing as strategy to treat atherosclerosis remains unknown.

An emerging target of interest is the epigenetic reader protein BRD4, which belongs to the bromodomain and extraterminal domain (BET) protein family and, through its binding affinity for acetylated histones, acts as a transcriptional coactivator during inflammation.^4^ Notably, in vivo studies using an atherosclerotic mouse model have demonstrated that BRD4 inhibitors can reduce plaque deposition by up to 50%.^5,6^ While inhibition of BRD4 using pharmacological mimics of acetylated histone 3 is known to suppress inflammation in macrophages,^7^ BRD4 is also widely implicated in proproliferative and prooncogenic roles.^8^ Within this latter context, BRD4 has been found to form complexes with the mechanosensitive transcriptional coactivators yes-associated protein (YAP) and its paralog transcriptional coactivator with PDZ-binding motif (TAZ), where it facilitates oncogenic transcriptional addiction in cancer cells.^9^ These mechanisms corroborate the known role of BRD4 as a scaffold that facilitates the opening of chromatin and assembly of transcriptional complexes, thereby priming regions of the genome for coordinated gene activation.^10^ However, the gene regulatory mechanisms underlying BRD4's involvement in atherosclerosis and its potential interaction with YAP/TAZ during disease progression have not been established.

YAP and TAZ are widely known for their proproliferative, antiapoptotic, and prooncogenic properties,^11^ but recent studies have revealed that their overexpression/activation contributes to proinflammatory pathologies characterized by persistent inflammation, fibrosis, and delayed tissue regeneration.^12^ Notably, macrophage YAP/TAZ activity has been recently reported to play adverse roles in atherosclerosis,^13^ nonalcoholic steatohepatitis,^14^ inflammatory bowel disease,^15^ and regeneration post-injury in the lungs,^16^ skin^17^ and myocardium.^18^ Our work has shown that YAP/TAZ are critical for macrophage mechanosensing and modulate their inflammatory response in response to changes in the stiffness of the microenvironment.^3^ Macrophages cultured in stiff environments or in contact with stiff biomaterials in vivo display enhanced YAP activity and inflammatory activation.^17^ Since YAP/TAZ cannot regulate transcription through direct chromatin interactions,^19^ and canonical chromatin binding partners of YAP/TAZ (namely TEAD1-4) are not typically associated with inflammation,^12,17^ we hypothesized that YAP/TAZ drive macrophage inflammation and, subsequently, aberrant lipid accumulation during atherogenesis through direct interaction with BRD4.

In this study, we aimed to investigate the role of BRD4-YAP/TAZ interactions in macrophage inflammation and lipid accumulation. We performed RNA-seq analyses on gain- and loss-of-function macrophage cell lines of YAP, TAZ, and BRD4 to elucidate their shared genetic targets and regulatory interdependence in the macrophage proinflammatory activation process. We found that YAP/TAZ and BRD4 activity all have profound effects on macrophage inflammation and lipid uptake receptors and postulated that YAP/TAZ and BRD4 may impact oxLDL uptake in macrophages. oxLDL uptake was hindered by YAP, TAZ, and BRD4 knockdown, and enhanced by ectopic activation of YAP/TAZ and in the context of inflammatory activation. Since YAP activation in macrophages has been shown to promote atherosclerosis,^13^ we hypothesized that BRD4 inhibition might confer atheroprotective effects by reducing YAP/TAZ activation, inflammation, excessive macrophage infiltration, and lipid accumulation. Testing this in an atherosclerosis mouse model, we find that BETi reduces plaque formation, the expression of proinflammatory and atherogenesis-associated target, as well as subdued the YAP/TAZ expression ie associated with atherogenesis. Together, these results highlight a new mechanism underlying the role of BRD4 in atherogenesis and support the potential for BETi as a treatment for cardiovascular disease.

## 2. Materials and methods

### 2.1 Cell culture, activation, and treatment conditions

THP-1 cells (ATCC Cat. TIB-202) were cultured in RPMI medium 1640 (ATCC formulation, Gibco Cat. A10491-01), with 10% fetal bovine serum (FBS), 50 µM β-mercaptoethanol, 100 U/mL penicillin, 100 µg/mL streptomycin, and differentiated into macrophages using 20 nM of phorbol 12-myristate 13-acetate (PMA) treatment for 48 h. This was followed by BETi (see details below) treatment for 24 h, when applicable. Bone marrow-derived macrophages (BMDMs) were obtained by differentiating bone marrow cells from the femur and tibia of 8 to 12-week-old female C57BL/6J mice (Jackson Laboratory) for 7 days using D10 media (DMEM with high glucose, 10% FBS, 100 U/mL penicillin, 100 µg/mL streptomycin, 292 µg/mL glutamine) containing murine macrophage colony-stimulating factor (10% of CMG14-12/L929 cell conditioned media) on nontreated culture plates. Bone marrow harvest was performed per Institutional Animal Care and Use Committee (IACUC) approved protocols established at the University of California, Irvine.

BET inhibitors used in the study are 0.5 µM iBET-762 (Molibresib, Sigma Cat. SML1272), 0.5 µM JQ1 (BPS Bioscience Cat. 27402), 0.5 µM ABBV-744 (Medchemexpress Cat. HY-112090), 5 µM RVX-208 (Apabetalone, Selleck Chemical Cat. S7295), 0.5 µM GSK046 (Medchemexpress Cat. HY136571), 0.5 µM GSK620 (Medchemexpress Cat. HY-137892), and 0.1 µM MZ1 (Cayman Chemical Cat. 21622). Unless otherwise specified, BETi henceforth refers to 0.5 µM ABBV-744 treatment for 24 h in vitro, and 5 mg/kg oral drug administration in vivo. Inflammatory stimulation was induced using 10 EU/mL ultrapure lipopolysaccharide (LPS, Invivogen Cat. tlrl-3pelps) and 10 ng/mL recombinant human interferon-gamma (IFNγ, Peprotech 300-02). BMDMs were polarized using 10 EU/mL ultrapure lipopolysaccharide and 10 ng/mL recombinant mouse interferon-gamma (IFNγ, R&D System Cat. 485MI100). High oxidized low-density lipoprotein (oxLDL, Kalen Biomedical Cat. 770252) was used at 20 µg/mL for 6 h or 24 h to induce foam cell formation, and DiI-oxLDL (Kalen Biomedical Cat. 770262) was used at 5 µg/mL for 6 h or 24 h to study oxLDL uptake. Experiments that activated macrophages using human inflammatory cytokines used 50 ng/mL recombinant TNF (Peprotech Cat. 300-01A), 50 ng/mL IL-6 (Peprotech Cat. 200-06), or 50 ng/mL IL-1β (Peprotech Cat. 200-01B) for 6 h.

Post experiments, cells were either fixed with 4% paraformaldehyde (PFA) for immunostaining and proximity ligation assay (PLA), lysed in Trizol (Sigma) for qRT-PCR, or lysed in RIPA buffer (VWR) with protease and phosphatase inhibitor cocktail (ThermoFisher) for protein analysis using Western blots. A minimum of 3 individual replicate experiments were performed for all analyses.

### 2.2 Lentiviral transduction and cell lines generation

THP-1 cells expressing shRNA targeting YAP, TAZ, and BRD4, and CMV promoter-mediated YAP^5SA^ and TAZ^S89A^ expression were generated by lentiviral spinfection. Lentiviral transfer plasmids for the shRNA were created using the RNAi consortium vector pLKO.1 (pLKO.1-TRC cloning vector was a gift from David Root, Addgene Cat. 10878).^20^ Each of the shYAP, shTAZ, and shBRD4 cells was created by cotransduction with viruses expressing 2 different shRNA against the targets (sequences provided in Extended Data Table S1). A shScram sequence was used to generate control cells. Lentiviral transfer plasmids for YAP^5SA^ and TAZ^S89A^ expression were created by modifying an empty backbone^21^ (pLenti-CMV-MCS-GFP-SV-puro was a gift from Paul Odgren, Addgene Cat. 73582) to express an SFB (S protein-FLAG-Streptavidin binding peptide) triple epitope tag, a T2A sequence, and an eGFP sequence on the C-terminal of the protein of interest. YAP^5SA^ was then migrated to this pLenti-CMV-MCS-SFB-T2A-eGFP from existing plasmids^22,23^ (pQCXIH-Myc-YAP-5SA was a gift from Kunliang Guan, Addgene Cat. 33093 and pLenti-EF-FH-TAZ S89A-ires-blast was a gift from Yutaka Hata, Addgene Cat. 52084). Control cells expressed the contents of the empty transfer vector.

Lentiviral particles were generated using transient expression in 293T cells. The 6 µg of transfer plasmids were mixed with 3 µg each of packaging plasmid psPAX2, and envelope plasmid pMD2.G (psPAX2 and pMD2.G were gifts from Didier Trono, Addgene Cat. 12260 and 12259 respectively), along with 36 µL of 1 mg/mL polyethyleneimine (PEI) per plate of 80% to 90% confluent 293T cells. The lentivirus from the supernatant was used to spinfect THP-1 cells with 10 µg/mL of hexadimethrine bromide (polybrene, Sigma) for 2 h at 2,500 RPM and 37 °C. The media was replaced after 24 h, and puromycin was added after 2 to 3 d of cell recovery for positive selection. Puromycin-selected cells were expanded, validated using qRT-PCR and Western blotting, and cryopreserved before further experimentation.

### 2.3 RNA isolation, qRT-PCR, and RNA-Seq

RNA was isolated from the cells or animal tissue using chloroform extraction, converted to cDNA using the high-capacity cDNA transcription kit (Thermo Fisher Cat. 4368814) and quantified using quantitative PCR (qPCR). Samples were analyzed using SsoAdvanced Universal SYBR Green Supermix (Bio-Rad Cat. 1725272). Primers were at 300 nM, and 2-step qPCR was performed according to the manufacturer's protocol. The primers used for qRT-PCR are described in Extended Data Table S2.

For RNA-Seq, cells were treated with 10 EU/ml LPS and 10 ng/mL IFNγ for 24 h. Before stimulation, cells were exposed to BETi for 24 h where applicable. RNA was isolated using a Trizol-free extraction kit (Takara Cat. 740955.5) from 3 separate experiments for RNA-Seq. UCI Genomics High Throughput Facility performed poly-A enrichment (stranded mRNA) library construction, and an average of 40 million paired-end reads were obtained per sample with Illumina NovaSeq6000. Adapters were trimmed with Cutadapt (v3.7),^24^ and reads were aligned using HISAT2 (v2.1.0)^25^ using hg38 assembly. Counts were calculated from the aligned reads using HTSeq (v0.11.2).^26^ Normalization and differential gene expression analysis were carried out on DESeq2 (v1.36.0)^27^ package on R (v4.2.0). Genes with a count per million of 2 or above in all 3 replicates within at least one condition were retained for DEG analysis. Genes were considered differentially expressed with a Benjamini−Hochberg correction adjusted *P*-value of 0.01 and |LFC|>2. Gene enrichment analyses were performed using ShinyGO (v0.77)^28,29^ and GSEA (v4.2.3).^30^

### 2.4 Immunocytochemistry staining

Immunocytochemistry (immunofluorescence) staining of in vitro samples was performed by fixation with 4% PFA (10 min), permeabilization with 0.3% Triton-X in PBS (10 min), blocking with 1% BSA (2 h), primary antibody staining (overnight, 4 °C), secondary antibody/Hoechst 33342/phalloidin (ThermoFisher Cat. A12379) staining (1 h) and mounting with Fluoromount-G (Southern Biotech). Primary antibodies used were anti-YAP (1:200, Santa Cruz Cat. sc-376830), anti-TAZ (1:500, Bioss Cat. bs-12367R), and anti-BRD4 (1:500, Abcam Cat. ab84776). Samples were visualized using a confocal microscope (Olympus Fluoview FV3000) and analyzed using Fiji software.^31^

### 2.5 Proximity ligation assay

Cells were cultured under indicated conditions on 8-well chamber slides (ThermoFisher Cat. 154534), fixed, permeabilized, blocked, and primary antibody stained using paired antigen targets for YAP (1:200, Santa Cruz Cat. sc-376830), TAZ (1:200, Santa Cruz Cat. sc-518026), or FLAG (1:400 Cell Signaling, Cat. 8146) (all mouse antibodies) with anti-BRD4 (1:400, Bethyl Cat. 50-156-1487) rabbit antibody. Proximity ligation and amplification were performed using a PLA kit (Sigma Cat. DUO92101-1KT), according to the manufacturer's instructions. Samples were visualized using a confocal microscope (Olympus Fluoview FV3000) using a 40 × oil immersion lens. Images captured were analyzed for the number of PLA spots in the nuclei using the SpotCounter plugin in Fiji.^32^

### 2.6 Co-immunoprecipitation

Whole-cell lysates were prepared using non-denaturing lysis buffer (20 mM Tris HCl pH 8, 137 mM NaCl, 10% glycerol, 1% NP-40, 2 mM EDTA) with protease and phosphatase inhibitor cocktail (ThermoFisher) and sonication (ice bath, 30 min). The 2 µg of anti-YAP, anti-TAZ, or anti-BRD4 antibodies were added per milliliter of lysate and incubated overnight with constant rotation at 4 °C. 25 µL of magnetic Protein A/G beads (ThermoFisher, Cat. 88802) were then added and the samples incubated for 2 h at room temperature. The beads were separated using a magnetic tube stand and washed 5 times using the lysis buffer. Subsequently, the immunocomplexes were eluted from the beads using 0.1 M glycine (pH 2.0), neutralized with Tris buffer (pH 8.0), and analyzed using Western blotting.

### 2.7 Western blot

For Western blotting, SDS-PAGE was performed using 4% to 15% gels (Bio-Rad) at 80 to 100 V, and the proteins were transferred using a dry blot transfer system to nitrocellulose membranes (iBlot2). The blot was blocked with 5% bovine serum albumin (BSA) in Tris-buffered saline with 0.1% Tween-20 (TBST) and probed overnight at 4 °C with either anti-YAP (1:1,000, Cell Signaling Cat. 14074), anti-TAZ (1:1,000, Cell Signaling Cat. 83669S), anti-FLAG (1:1,000, Cell Signaling Cat. 8146), anti-BRD4 (1:1,000, Abcam Cat. ab84776), anti-CD36 (1:1,000, Cell Signaling Cat. 14347S), or anti-OLR1 (1:1,000, Abcam Cat. ab60178) antibodies. GAPDH antibody (1:2,000, Santa Cruz, Cat. sc-59540) was used to normalize the cell lysate loading. After 3 × washes using TBST, secondary antibodies conjugated to HRP were added at 1:2,000 dilution in 5% BSA and incubated at RT for 1 h. After 3 × washes with TBST, the blot was developed using SuperSignal West Femto substrate (ThermoFisher Cat. 34096), and chemiluminescence was visualized using a GelDoc (Bio-Rad).

### 2.8 Adeno-associated virus preparation

Liver promoter-driven adeno-associated virus (AAV) plasmids containing the gain-of-function mutant form of murine proprotein convertase subtilisin/kexin type 9 (PCSK9) and luciferase (control) were obtained from Addgene (pAAV/D377Y-mPCSK9 was a gift from Jacob Bentzon, Addgene Cat. 58376; pAAV-TBG-FFLuc was a gift from Phillip Zamore, Addgene Cat. 35658).^33^ Shuttle plasmids and AAV helper plasmids pAdDeltaF6 (pAdDeltaF6 was a gift from James M. Wilson, Addgene Cat. 112867) and pAAV2/9n (pAAV2/9n was a gift from James M. Wilson, Addgene Cat. 112865) were co-transfected into AAVpro 293T cells (Takara Cat. 632273) using PEI, following Addgene's protocol, to produce serotype 9 AAV. Both media supernatants and cells were collected 5 d after transfection and processed separately. AAV from supernatant was precipitated using polyethylene glycol in 0.2 M NaCl for 4 h at 4 °C, and then centrifuged for 30 min at 2,500 *g*, 4 °C. Cells were resuspended into a lysis buffer (PBS with 0.001% Pluronic F-68, 200 mM NaCl) and sonicated using 4 × 1 s pulses with at least 15 min on ice between each pulse at 50% amplitude (Branson Sonifier SFX250). Supernatant from the cell lysate and the AAV pellet from precipitated supernatant were combined and digested using benzonase (50 U/mL) for 45 min, 37 °C. The virus was purified and concentrated using the AAVpro concentrator (Takara, Cat. 6674) per the manufacturer's protocol. The purified virus was aliquoted and stored at −80 °C prior to use. qPCR was used to quantify the virus in terms of vector genome copies (VG). The respective AAV plasmids (plasmid copies of 10^3^ to 10^9^) were used as standards against which serially diluted viral solutions (10^3^ to 10^7^-fold dilutions) were compared to calculate the viral titer.

Human YAP^5SA^ (from pQCXIH-Myc-YAP-5SA) and TAZ^S89A^ (from pLenti-EF-FH-TAZ S89A-ires-blast) genes were migrated into AAV backbone plasmid pAAV-TBG-FFLuc, replacing the TBG promoter with the mouse *Lyz2* promoter region (Sequence: AAG GAG GGA CTT GGA GGA TGC TTA AAT AGC AGG CAT GCT TTC TCT AGT CAG CCA GCA GCT), and replacing the luciferase gene with human YAP-5SA and TAZ-S89A mutant genes using standard digestion and ligation methods. A 50-50 mix of serotypes 2 and 6 viruses encoding human YAP5SA and TAZS89A was used to overexpress YAP and TAZ in myeloid cells in mice. AAV preparation for AAV2/6-Lyz2-YAP^5SA^ and AAV2/6-Lyz2-TAZ^S89A^ was carried out similarly to the abovementioned method. The helper plasmids used here were pAAV2/2 (gift from Melina Fan, Addgene Cat. 104963) and pRepCap6 (gift from David Russell, Addgene Cat. 110770)^34^ in a 50-50 mix, in addition to pAdDeltaF6.

### 2.9 Atherosclerosis mouse model

The atherosclerosis model implemented in this study was performed per Institutional Animal Care and Use Committee (IACUC) approved protocols established at the University of California, Irvine. To induce atherosclerosis, wild-type male C57BL/6J mice were injected with 2 × 10^12^ VG of AAV9-D377Y-mPCKS9 (henceforth AAV-PCSK9) through the tail vein as described before.^35^ Control injections of 2 × 10^12^ VG of AAV9-Luc (henceforth AAV-Luc) were provided to animals during a pilot study to establish the model. For studies involving YAP and TAZ activation, AAV-PCSK9 animals were also co-injected with 1 × 10^13^ VG AAV2/6-Lyz2-YAP^5SA^ and AAV2/6-Lyz2-TAZ^S89A^ virus each (henceforth AAV-YT) to overexpress gain-of-function versions of human *YAP* and human *TAZ* under the control of the *Lyz2* promoter, resulting in their expression in myeloid cells. Thirty days after the injection, the animals were fed a high-fat diet ad libitum (16% fat and 1.25% cholesterol by wt., Research diets Cat. D12336) for 60 d (90 d in case of the initial pilot study that compared the effects of AAV-PCSK9 injections to control AAV-Luc). During this period, animals were weighed daily and orally dosed with 5 mg/kg ABBV-744 dissolved in 10% DMSO in corn oil (BETi) *q.d.* using a 22-G curved gavage needle. Corn oil with 10% DMSO was used as a control. At the end of the study, animals were sacrificed, and blood was collected by cardiac puncture. Hearts were perfused with PBS to clear the aorta and were then dissected out.^36^ The perivascular adipose tissue was removed by microdissection. The aorta tissue was either fixed in formalin (for *en face* Oil Red O staining) or dissected into smaller sections that were embedded in optimal cutting temperature medium (OCT, for IHC/IF), dissociated into cells (for flow cytometry), or homogenized in Trizol (for qRT-PCR). Of the 12 animals treated in each group, 3 to 5 animals each were analyzed for each of the en face staining, cross-sectional histology, and flow cytometry analyses. In addition, serum analyses were done on all test animals.

### 2.10 Oil red O staining

Aorta tissue was cleared of perivascular adipose and fixed in formalin for 24 h. After washing with PBS, the aorta was carefully sliced open and then stained for 20 min. A working solution of Oil Red O was prepared by diluting 3 parts stock (0.5% Oil Red O in isopropanol) with 2 parts deionized water and filtering it. The aorta was quickly rinsed in 60% isopropanol, washed with PBS several times, then mounted on wax dishes and imaged. *En face* Oil Red O staining on 15 mm of aorta tissue (arch) proximal to the heart was quantified using Fiji. Oil Red O staining in vitro was performed similarly and was followed by hematoxylin staining and mounting. To quantify the Oil Red O stain in vitro, accumulated stains within cells were extracted in isopropanol for 5 min and read using a plate reader (Bio-Rad) at 490 nm.

### 2.11 Serum cholesterol estimation

The collected blood serum was analyzed for total cholesterol, LDL, and HDL levels using a cholesterol estimation kit per the manufacturer's recommendations (Sigma Cat. MAK043).

### 2.12 Enzyme-linked immunosorbent assay (ELISA)

Levels of the inflammatory cytokines IL-6, IL-1β, and MCP-1 were measured in serum using mouse ELISA kits (BioLegend Cat. 431301, 432604, and 432704) according to the manufacturer's instructions.

### 2.13 Flow cytometry

Aorta tissue was dissociated into single cells by digesting in an enzyme cocktail—Aorta Dissociation Enzyme Stock Solution (Collagenase Type XI: 1 mg/mL, Hyaluronidase Type 1-S: 0.1 mg/mL, DNAase I: 0.2 mg/mL, collagenase I: 1 mg/mL) as described before.^37^ The cells were passed through a 70 µm cell strainer, cell debris removed (Miltenyi Biotec, Cat. 130-109-398), fixed using 4% PFA (15 min), permeabilized using 90% ice-cold methanol (30 min), blocked using anti-CD16/CD32 antibody (Biolegend Cat. 101319), and subsequently co-labeled with anti-CD45-FITC (1:50, Biolegend Cat. 157214) and anti-CD68-APC (1:50, Biolegend Cat. 137007) antibodies. YAP (1:50, Proteintech Cat. 13584-1-AP) and TAZ (1:50, Bioss Cat. bs-12367R) were labeled by incubation using primary antibody overnight, followed by donkey anti-rabbit-PE secondary antibody staining for 1 h (1:200, Biolegend Cat. 406421). Appropriate concentration-matched isotype (Biolegend Cat. 400511 and 400605), secondary antibody-only, and cells-only controls were utilized. Flow cytometry on DiI-oxLDL labeled cells was also performed by scraping cells into ice-cold cell dissociation buffer (Gibco), fixation, debris removal, staining, and cytometry. Blood cells from mice were analyzed by ACK lysis, fixation, and staining, followed by cytometry as described above. Flow cytometry was performed on an LSR-II system (BD), and data were analyzed on FlowJo software (BD).

### 2.14 Immunohistochemistry and immunofluorescence

Mouse aorta tissue was embedded in OCT compound and stored at −80 °C before sectioning using a cryostat to generate 7 µm slices. The sections were stored at −80 °C until further processing. The samples were thawed and fixed using 4% PFA for 15 min and washed 4 × with PBS. Hematoxylin and Eosin (H&E) staining was performed per the manufacturer's recommendations (Abcam Cat. ab245880). Fixed sections were stained for 20 min in Oil Red O working solution, followed by a quick rinse in 60% isopropanol, multiple washes with PBS, and mounted. The sections were permeabilized using 0.1% Triton X-100 for 10 min for immunofluorescence staining. After 3 × wash with PBS containing 0.1% Tween 20 (PBST), sections were blocked with 5% rabbit serum in PBST for 2 h. Primary antibody staining was done overnight for F4/80 (1:500, Cell Signaling Cat. 71299), YAP (1:200, Santa Cruz Cat. sc-376830), TAZ (1:200, Santa Cruz Cat. sc-518026), ICAM-1 (1:200, Santa Cruz Cat. sc-8439), VCAM-1 (1:200, Santa Cruz Cat. sc-13160), CD36 (1:400, Cell Signaling Cat. 14347), or LOX1 (1:400, Abcam Cat. ab60178). After 3 × wash with PBST, secondary staining was done for 1 h using 1:1,500 diluted anti-rat (Abcam Cat. ab150157), anti-mouse (Abcam Cat. ab150076), anti-rabbit (Abcam Cat. ab150108), or anti-goat (Abcam Cat. ab150132) antibodies, in addition to nuclear stain with 1:2,000 Hoechst 33342. Stained tissue sections were viewed using a Keyence BZ-X810 microscope and analyzed using Fiji software.^32^

### 2.15 Chromatin immunoprecipitation quantitative PCR (ChIP-qPCR)

ChIP experiments were performed using previously established methods.^38^ Between 15 and 20 million cells were plated for each of the conditions. After 2 h of inflammatory stimulation, culture media was aspirated, and the cells were crosslinked with 1% PFA (10 min). All subsequent steps were performed on ice. The samples were quenched with 0.125 M glycine in PBS for 5 min, and cells were scrapped into cell lysis buffer (0.5% NP-40 and 85 mM KCl in 20 mM Tris-HCl, pH 8.0). Nuclei were pelleted at 2,500 *g* for 5 min and then lysed with nuclei lysis buffer (0.1% sodium dodecyl sulfate, 0.5% sodium deoxycholate, 1% Nonidet-40 in 10 mM Tris-HCl, pH 7.5). Chromatin from the nuclear lysate was then sheared for 8 min (0.7 s on and 1.3 s off pulses) using a probe sonicator (Branson Sonifier SFX250) at 40% amplitude. Approximately 10 µg of chromatin was used per immunoprecipitation with 2 µg of H3Ac (Millipore Sigma, Cat. 06-599), FLAG (Cell Signaling, Cat. 14793), or BRD4 antibodies (Bethyl Cat. 50-156-1487) at 4 °C overnight. Rabbit IgG was used as a control for immunoprecipitation. Immunoprecipitated complexes were recovered with magnetic protein A/G beads and washed twice serially with low salt immunocomplex buffer, high salt immunocomplex buffer, lithium chloride immunocomplex wash buffer, and Tris-EDTA (pH 8). Immunoprecipitated protein-DNA complexes were eluted into 1% SDS and 100 mM sodium bicarbonate solution, then reverse crosslinked overnight at 65 °C in a solution containing RNase A and proteinase K. The DNA was recovered using AMPure XP magnetic beads (Beckman Coulter), washed with 70% ethanol, and then quantified by qPCR using primers listed in Extended Data Table S3. DNA enrichment has been represented as the fraction of input and is presented as fold change relative to bound protein signals in unstimulated macrophages without BETi.

## 3. Results

### 3.1 YAP and TAZ induce proinflammatory gene activity that is reduced upon BETi treatment in macrophages

**Fig. 1.**
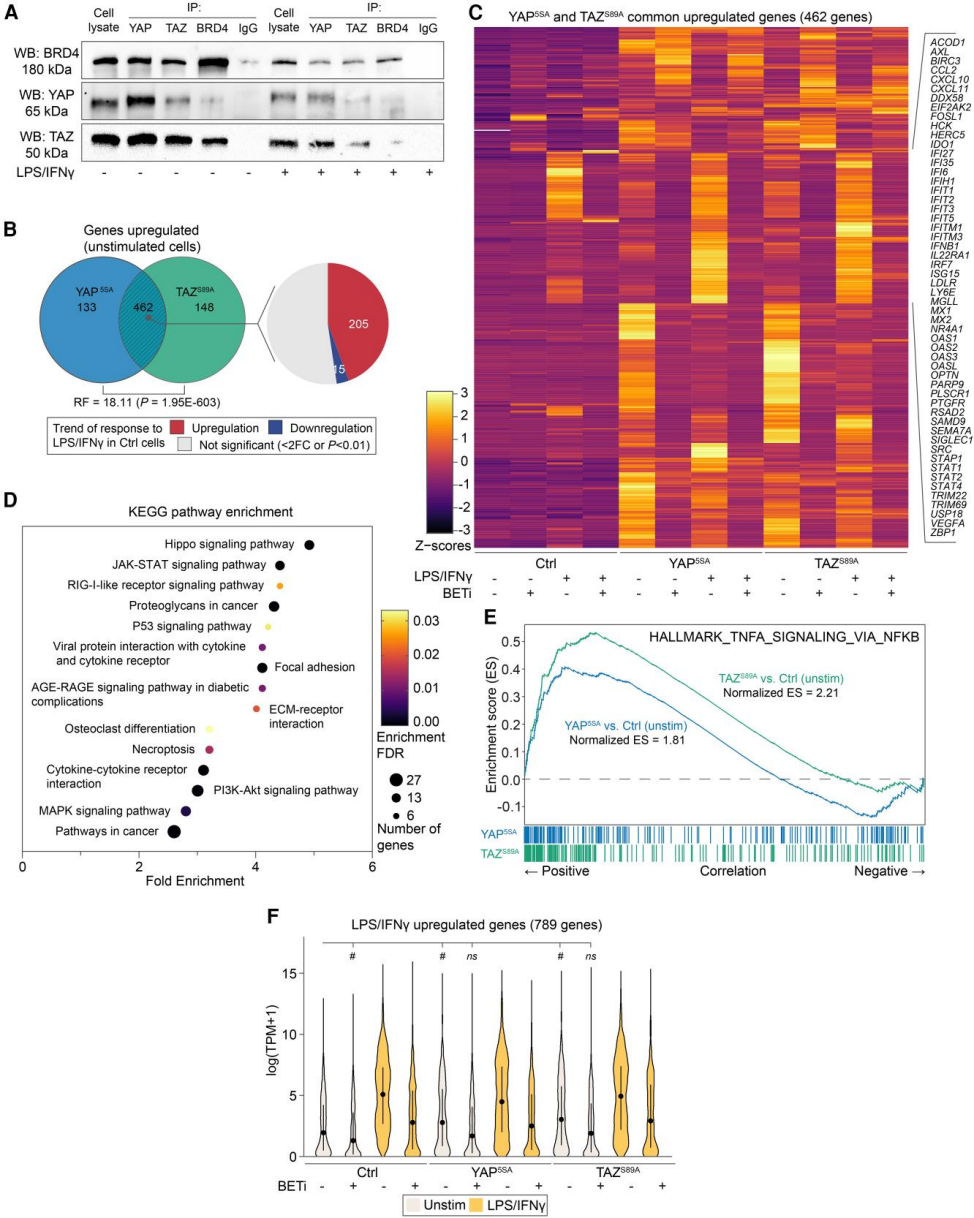
YAP and TAZ overexpression induce inflammation in macrophages that can be curbed with BETi. (A) YAP, TAZ, and BRD4 co-immunoprecipitation in THP-1 derived macrophages under both unstimulated and LPS/IFNγ-stimulated conditions. (B) Overlaps between the genes upregulated in unstimulated cells with YAP^5SA^ and TAZ^S89A^ expression in comparison to the empty vector control (Ctrl) unstimulated cells, and a break-down of the common YAP^5SA^ and TAZ^S89A^ upregulated genes with respect to inducibility in control cells with LPS/IFNγ treatment. (C) Gene expression in the common YAP^5SA^ and TAZ^S89A^ upregulated genes ± LPS/IFNγ stimulation ± BETi treatment. (D) Top 15 KEGG pathway enrichment terms in the common YAP^5SA^ and TAZ^S89A^ upregulated genes. (E) Increase enrichment for hallmark TNFA signaling via NF-κB identified by GSEA in YAP^5SA^ and TAZ^S89A^ expressing cells (ES: Enrichment score). (F) Gene expression in the LPS/IFNγ-induced genes with YAP^5SA^ and TAZ^S89A^ expression and BETi treatment. Violin plots show quartiles and median. # denotes *P* < 0.05 in paired Friedman test with Dunn's post-hoc for multiple comparisons. Venn diagram representation factor (RF) and *P*-value are based on the under- or over-enrichment of gene representation based on the cumulative distribution function (CDF) of their hypergeometric distribution.

We evaluated the expression of YAP and TAZ in human macrophages derived from THP-1 cells using PMA, and found upregulation of YAP and stable TAZ expression upon differentiation, consistent with our previous studies in primary human macrophages^17^ (Fig. S1a–c). Thus, we used this model cell line to probe whether the YAP/TAZ/BRD4 trio could directly interact to form complexes within macrophages. Co-immunoprecipitation assays confirmed the interaction between YAP and TAZ, and revealed that YAP and TAZ were both bound with BRD4 (Fig. 1A). Moreover, stimulation of macrophages with inflammatory agonists LPS and IFNγ did not appear to affect complex formation.

Given that YAP and TAZ have been implicated in several inflammatory diseases, we next hypothesized that YAP/TAZ-mediated transcriptional activity could be directly linked to inflammation in a BRD4-dependent manner. To test this hypothesis, we created THP-1 cell lines that overexpressed constitutively active (through phosphorylation-resistant mutations) forms of YAP and TAZ (henceforth referred to as YAP^5SA^ and TAZ^S89A^) to understand their effects on inflammation through RNA-Seq (Fig. S2a, b). Phosphorylation of YAP/TAZ results in its cytoplasmic sequestration and eventual degradation. Given that both mutants are resistant to phosphorylation by LATS1/2 (their upstream effectors of the Hippo pathway), YAP^5SA^ and TAZ^S89A^ fail to degrade and remain localized to the nucleus, resulting in increased transcriptional activity of target genes.^39,40^ Transducing THP-1 cells with an empty vector (Ctrl) generated a control cell line.

RNA-seq analysis of YAP^5SA^ and TAZ^S89A^ cells reveals significant commonalities in their transcriptomes, irrespective of the polarization state of the macrophages (Fig. 1B, Fig. S2c–e). We found that more than 75% of YAP^5SA^-upregulated genes were common with TAZ^S89A^ cells and vice versa in the unstimulated condition, suggesting redundant roles of YAP and TAZ. As expected, canonical YAP/TAZ targets such as *CYR61*, *CTGF*, *ANKRD1*, *PLAU*, *FOSL1*, and *SERPINE1* were overexpressed in macrophages expressing YAP^5SA^ and TAZ^S89A^ (Fig. S2f). However, we also found a large number of genes upregulated in YAP^5SA^ and TAZ^S89A^ macrophages that were novel (ie not identified in previous studies on cancer cell lines such as MDA-MB-231^41^ and transformed MCF-10A cells^42^). A significant number of YAP^5SA^ and TAZ^S89A^-upregulated genes are genes that are induced in control macrophages upon inflammatory stimulation through LPS/IFNγ treatment (Fig. 1C). More specifically, over 44% of genes upregulated by the overexpression of constitutively active YAP/TAZ (under unstimulated conditions) are upregulated in Ctrl cells upon LPS/IFNγ stimulation compared to 3% genes that were downregulated in Ctrl cells upon inflammatory stimulation, suggesting that YAP/TAZ activation primarily upregulates genes involved in establishing a proinflammatory phenotype in macrophages. These genes include proinflammatory cytokines and chemokines (*IFNB1*, *CCL2*, *CXCL10*, *CXCL11*, *CXCL14*, *VEGFA*), signaling kinases (*SRC*, *HCK*), and transcription factors (*STAT1*, *STAT2*, *STAT4*, *FOSL1*, *NR4A1*). Mapping the genes upregulated by YAP/TAZ to cell signaling pathways using KEGG pathway enrichment analysis, we found both expected pathways related to cell proliferation (Hippo, P53, and PI3K/AKT signaling and other cancer-related pathways), and novel signaling pathways related to immune regulation (JAK/STAT and MAPK pathways, viral response, cytokine response pathways), and mechanotransduction (focal adhesions and ECM−receptor interactions) (Fig. 1D). In addition, gene set enrichment analysis (GSEA) revealed that genes associated with inflammatory and fibrosis pathways, such as TNF signaling through NF-κB, hallmark IFNα, IL6-JAK-STAT3, TGF-β, P53 signaling pathways, and hypoxia response, are enhanced in macrophages expressing YAP^5SA^ and TAZ^S89A^ (Fig. 1E, Fig. S2g–k).

We next examined the effect of BET inhibition on the expression of inflammatory genes induced by YAP^5SA^ and TAZ^S89A^ overexpression. BET inhibitors have previously been shown to exert anti-inflammatory effects in classically stimulated macrophages.^7,43^ This observation holds across 7 different BRD4/BET pharmacological inhibitors that we tested at noncytotoxic concentration ranges, where BET inhibition reduced production of the proinflammatory cytokine MCP-1 in THP-1 macrophages within both stimulated (with LPS/IFNγ) and unstimulated conditions (Fig. S3a, b). For the remainder of the study (unless noted otherwise), we used BRD4/BET inhibitor ABBV-744 (henceforth BETi) as our BET inhibitor of choice, as it was an orally active clinical-stage drug that also consistently exhibited anti-inflammatory effects in vitro. Macrophages exhibited a subdued inflammatory response to LPS/IFNγ stimuli when treated concomitantly with BETi (Fig. 1F). Furthermore, the repressive effects of BETi treatment on gene expression were also observed more broadly across gene targets shared by both YAP^5SA^ and TAZ^S89A^ (Fig. 1C). Nuclear translation of YAP and TAZ was increased in macrophages upon treatment with LPS/IFNγ and inflammatory cytokine TNF-α (Fig. S4a, b), consistent with various studies showing increased nuclear translocation and activity of YAP/TAZ in inflamed cells (reviewed in^12^). Reportedly, BRD4 inhibition does not cause reduced YAP or TAZ expression in cancer cells.^9^ In agreement, we observed nonsignificant dynamics in YAP/TAZ expression and nuclear translocation in macrophages treated with BETi, within both stimulated and unstimulated conditions (Fig. S4c, d), suggesting that the anti-inflammatory activity of BETi treatment does not require modulation of YAP/TAZ levels or nuclear localization. Taken together, these results suggest that the active YAP/TAZ immunocomplexes work to orchestrate inflammatory programs in macrophages in a manner that can be subdued through pharmacological inhibition of BRD4.

### 3.2 BRD4, YAP, and TAZ perform interdependent roles in macrophage inflammatory gene target regulation

**Fig. 2.**
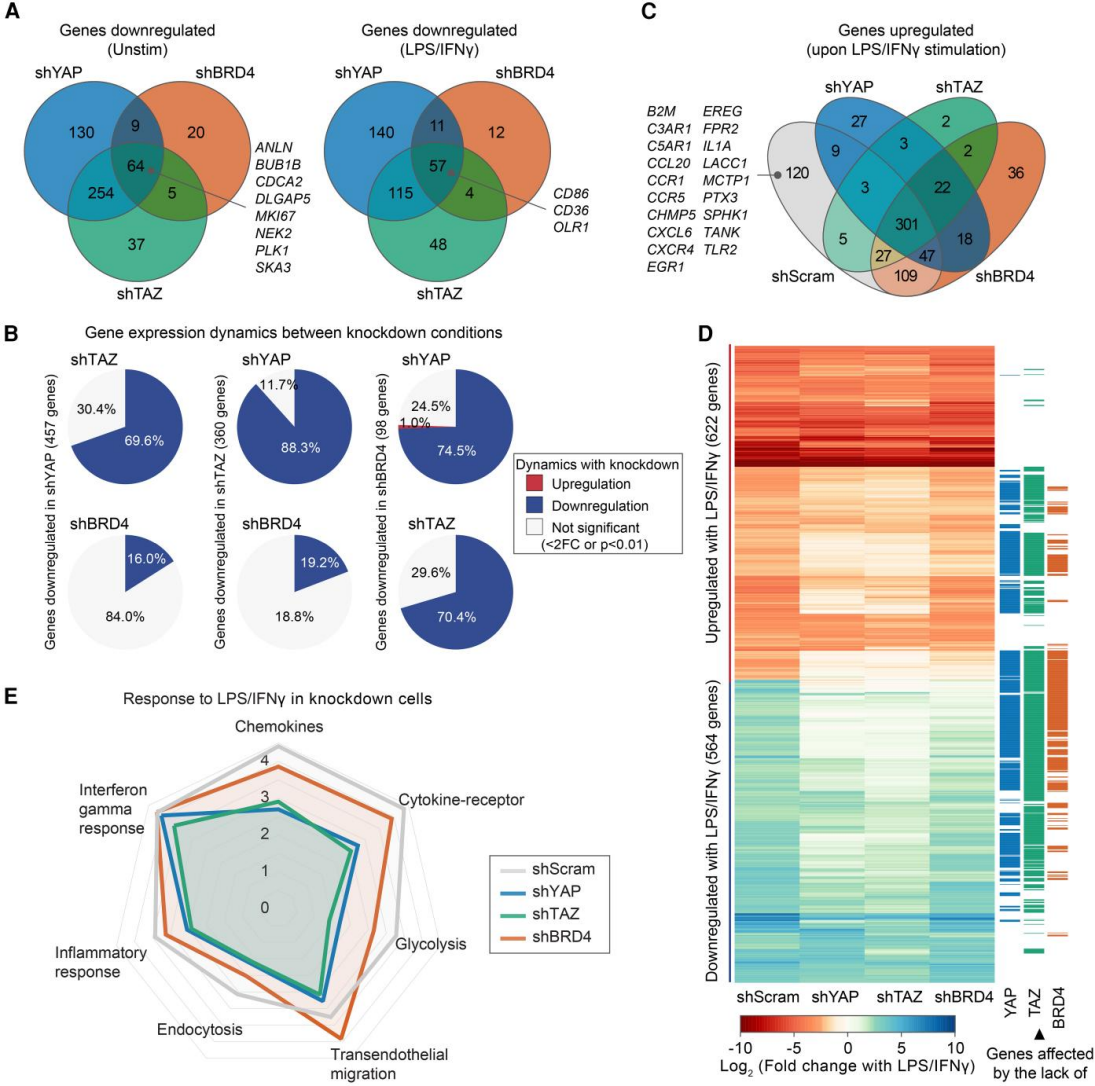
The loss of YAP, TAZ, and BRD4 causes overlapping anti-inflammatory effects in macrophages. (A) Overlap between the genes that are downregulated in shYAP, shTAZ, and shBRD4 expressing cells in comparison to shScram controls, in both unstimulated and LPS/IFNγ-stimulated cells. (B) LPS/IFNγ stimulation-induced gene dynamics in shScram, shYAP, shTAZ, and shBRD4 cells. (C) Genes upregulated with knockdown conditions upon LPS/IFNγ treatment. (D) Differential gene expression with LPS/IFNγ treatment in shScram, shYAP, shTAZ, and shBRD4 cells. (E) Response of knockdown cells to LPS/IFNγ.

Genetic silencing of YAP, TAZ, and BRD4 has each been known to have anti-inflammatory effects in classically activated macrophages.^17,43^ However, their interdependence in activating shared immune-regulated gene targets has not been explored. To test the interdependence of YAP, TAZ, and BRD4 during macrophage inflammatory gene activation, we inhibited the endogenous expression of each gene independently in THP-1 cells using lentiviral delivery of shRNA sequences targeting each gene (Fig. S5a). We then performed RNA sequencing on THP-1 cells from each knockdown condition, including a scramble shRNA for control, across both stimulated (with LPS/IFNγ) and unstimulated conditions. RNA-seq showed that a loss of endogenous YAP or TAZ expression through shRNA knockdown led to the downregulation of several overlapping gene targets in both stimulated and unstimulated conditions (∼46% and ∼63%, respectively), confirming a shared loss-of-function in THP-1 cells (Fig. 2A, Fig. S5b and c). Notably, genes downregulated in either the shYAP, shTAZ, or shBRD4 conditions were also significantly downregulated in the other 2 respective knockdown conditions (Fig. S5d). However, the loss of BRD4 expression was, by far, primarily associated with the downregulation of genes that were also downregulated in both YAP and TAZ knockdown conditions. Indeed, genes downregulated by shYAP and shTAZ accounted for 74.5% and 70.4% of gene targets downregulated by the loss of BRD4, respectively (Fig. 2B). In addition, the shBRD4 condition showed fewer differentially expressed genes in total compared to shYAP and shTAZ conditions, further suggesting BRD4's impact on gene regulation is primarily performed in tandem with YAP and TAZ. We also noted that genes commonly downregulated by YAP, TAZ, and BRD4 knockdowns in both stimulated and unstimulated conditions were predominantly involved in biological processes associated with cell division (Fig. 2A, Fig. S5e), pointing to the roles of YAP, TAZ, and BRD4 in macrophage survival and proliferation. Genes that govern mitosis (including *ANLN*, *BUB1B*, *CDCA2*, *DLGAP5*, *MKI67*, *NEK2*, *PLK1*, *SKA3*) are downregulated commonly by the absence of YAP, TAZ, and BRD4 irrespective of polarization state. In addition, we also noted that *CD86* (an activation marker), and *OLR1* and *CD36* (both oxLDL uptake receptors) were downregulated commonly in shYAP, shTAZ, and shBRD4 cells upon LPS/IFNγ stimulation. These data demonstrate the interdependence of YAP, TAZ, and BRD4 proteins in the gene activation of macrophages.

Interestingly, reduced YAP, TAZ, and BRD4 eliminated the ability of macrophages to regulate a common set of genes otherwise dynamically regulated by stimulation with LPS/IFNγ. Specifically, 120 genes upregulated in response to LPS/IFNγ across control (shScram) conditions (and 185 downregulated genes) were not upregulated (or downregulated) in either of the shYAP, shTAZ, or shBRD4 conditions (Fig. 2C). These include genes for proinflammatory cytokines/chemokines (*IL1A*, *CXCL6*, *CCL20*), receptors (*CCR1*, *CCR5*, *CXCR4*, *C3AR1*, *C5AR1*, *TLR2*), and signaling mediators (*TANK*). However, we did observe several genes that remained responsive to stimulation across all shRNA conditions (eg 301 upregulated and 140 downregulated genes), suggesting the impact of targeted knockdowns on genes dynamically regulated by LPS/IFNγ was heavily localized to specific gene subsets (Fig. 2D). Additionally, activation of gene functional categories associated with critical macrophage functions (such as cytokine-receptor interactions, endocytosis, chemokine production, glycolysis and the inflammatory response) were subdued across all knockdown conditions across stimulated and unstimulated conditions (Fig. 2E). Genes associated with IFNγ response, however, were not impacted. Notably, BRD4 knockdown appeared to upregulate genes associated with transendothelial migration (or diapedesis), consistent with our previous observations on the impact of iBET on macrophage migration.^43^ Taken together, a convergent loss of response to LPS/IFNγ treatment occurs in the YAP, TAZ, and BRD4 knockdown macrophages, underlining the importance of these proteins to macrophage inflammatory processes and their interdependence.

### 3.3 Macrophage uptake of oxLDL is influenced by YAP/TAZ activation and curtailed by BETi

**Fig. 3.**
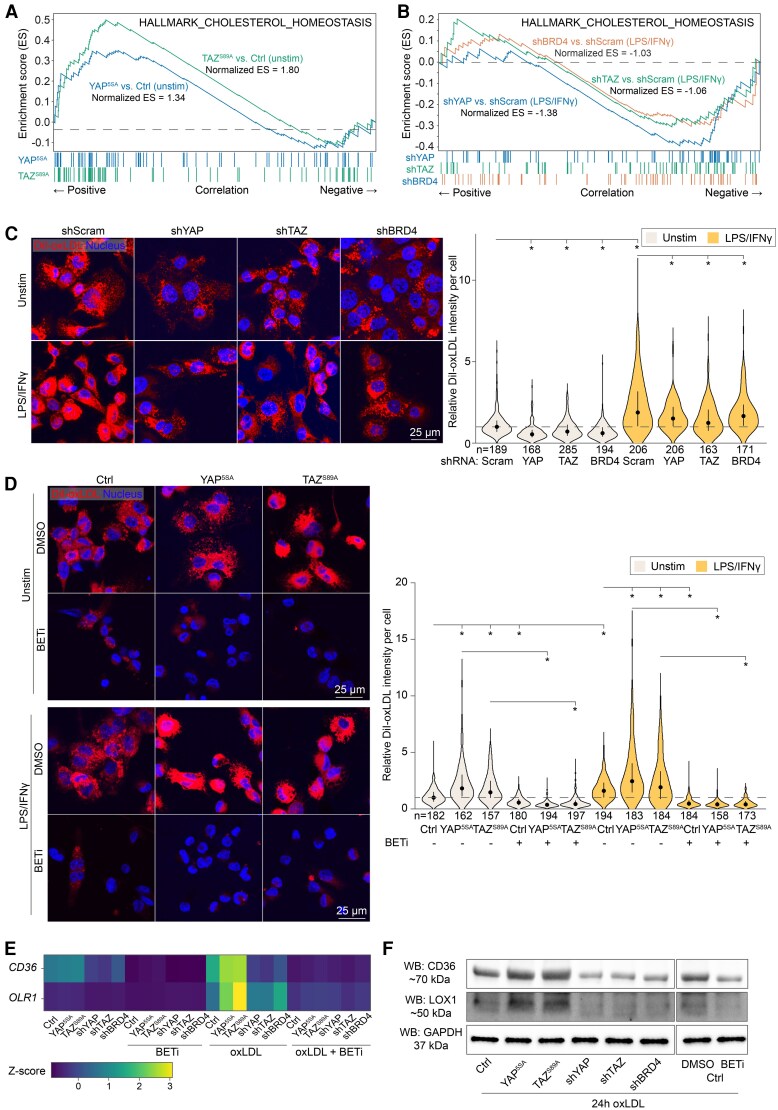
Macrophage oxLDL uptake is modulated by YAP, TAZ, and BRD4 expression. (A) GSEA analysis of hallmark cholesterol homeostasis genes identified by GSEA in YAP^5SA^ and TAZ^S89A^ expressing cells. (B) GSEA analysis of hallmark cholesterol homeostasis genes identified by GSEA in shYAP, shTAZ, and shBRD4 expressing cells under LPS/IFNγ stimulation. (C) Representative images (left) and quantification (right) of DiI-oxLDL uptake at 24 h in knockdown cells under unstimulated and LPS/IFNγ-stimulated conditions. (D) Representative images (left) and quantification (right) of DiI-oxLDL uptake at 24 h in YAP^5SA^ and TAZ^S89A^ expressing cells under unstimulated and LPS/IFNγ-stimulated conditions. (E) *CD36* and *OLR1* gene expression with perturbations in YAP, TAZ, and BRD4, measured by qRT-PCR in foam cells induced by oxLDL exposure. (F) CD36 and LOX1 protein expression with perturbations in YAP, TAZ, and BRD4, in oxLDL-treated cells. Violin plots show quartiles and median. *denotes *P* < 0.05 in 2-tailed unpaired Mann−Whitney *U* test.

Screening the effects of YAP/TAZ activation on macrophage functions, we find that fatty acid metabolism, especially cholesterol homeostasis, depended on YAP/TAZ activity. GSEA revealed that YAP^5SA^ and TAZ^S89A^ overexpressing cells exhibited enhanced expression of genes associated with cholesterol homeostasis relative to control cells in unstimulated conditions (Fig. 3A). Conversely, cells expressing shYAP, shTAZ, and shBRD4 were depleted in these genes under LPS/IFNg stimulation when compared to shScram control cells (Fig. 3B). Indeed, lipid uptake receptors, such as *OLR1* (gene encoding LOX1) and *CD36*, were among the genes downregulated commonly in YAP, TAZ, and BRD4 knockdown cells (Fig. 2A). YAP activity in macrophages^13^ and endothelial cells^44–46^ has also been implicated in atherosclerosis. Given that lipoprotein uptake is associated with proinflammatory activation of macrophages,^2^ and oxLDL is critical in inducing macrophages into foam cells and contributing to atherosclerosis, we hypothesized that oxLDL uptake might also depend on robust YAP, TAZ, and BRD4 expression. We found that cells expressing shYAP, shTAZ, and shBRD4 showed significantly reduced oxLDL uptake (Fig. 3C). LPS/IFNγ pretreatment is known to increase the expression of oxLDL uptake receptors in macrophages.^47,48^ In agreement, we find higher oxLDL uptake in macrophages pre-stimulated with these inflammatory agonists. Interestingly, even in stimulated cells, we find that a lack of YAP, TAZ, or BRD4 significantly reduced the uptake of oxLDL in macrophages (Fig. 3C, Fig. S6a).

We next tested whether expression of the constitutively active YAP^5SA^ and TAZ^S89A^ in macrophages would increase oxLDL uptake and whether subsequent BETi treatment could curb heightened uptake levels induced through aberrant YAP/TAZ overactivity. Indeed, we found that YAP^5SA^ and TAZ^S89A^ cells increased oxLDL uptake levels, which were attenuated with BETi treatment (Fig. 3D). oxLDL uptake measured by Oil Red O staining captured similar trends (Fig. S6b, c). We next explored if other BRD4 inhibitors could reduce macrophage uptake of oxLDL. Notably, all 7 BET inhibitors we tested significantly reduced oxLDL uptake in macrophages, independent of LPS/IFNγ treatment (Fig. S7a, b). We performed the same experiment in murine BMDMs, identifying similar oxLDL uptake reduction in cells exposed to various BRD4 inhibitors (Fig. S7c). We also observed that *CD36* and *OLR1* were upregulated by YAP/TAZ overactivation in macrophages, particularly under exposure to oxLDL (Fig. 3E). Remarkably, BETi treatment reduced the upregulation of *CD36* and *OLR1* mRNA, which was also confirmed at the protein level (Fig. 3F). This increased CD36 and LOX1 expression may be tied to increased YAP and TAZ nuclear translocation observed in foam cells pre-stimulated with LPS/IFNγ before oxLDL treatment (Fig. S4a, b).

We explored if direct interactions between YAP/TAZ and BRD4 were dynamic in macrophages undergoing inflammatory activation or exposure to oxLDL. Proximity ligation assays were performed to visualize in situ interactions of YAP and TAZ with BRD4. We found that interactions between YAP, but not TAZ, and BRD4 increase with LPS/IFNγ stimulation (Fig. S8a, b). Exposure to oxLDL increased BRD4 interactions with YAP and TAZ within both stimulated and unstimulated conditions. Remarkably, BETi treatment, known to interfere with BRD4 interactions with the chromatin, reduced interactions between YAP and TAZ (Fig. S8a, b). Thus, inflammatory stimulation concomitant with oxLDL exposure caused increased CD36 and LOX1 expression in macrophages in a YAP/TAZ/BRD4-dependent manner.

### 3.4 Atherosclerosis displays increased macrophage infiltration and YAP/TAZ expression

Earlier studies have shown that BRD4 antagonism can attenuate atherogenesis by up to 50%,^5,6^ but the mechanisms underlying their atheroprotective function remain largely unexplored. We hypothesized that BETi treatment could alleviate atherosclerotic disease by a combination of lower oxLDL uptake and reduced inflammation in macrophages that would otherwise facilitate the formation of foam cells. To test this, we used an AAV-based atherosclerosis mouse model that we validated first in a pilot study (Fig. S9a, b).^35^ Briefly, 30-day-old mice were injected with AAV-PCSK9 that triggered the overexpression of a murine proprotein convertase subtilisin/kexin (*PCSK9*) gain-of-function mutant in the liver. This causes hypercholesterolemia in these mice when fed a high-fat diet (HFD), owing to low-density lipoprotein receptor (LDLR) depletion in the liver and the consequent reduced fat uptake. We found that 3 mo of HFD resulted in mature plaque formation with plaque coverage of 40% in the AAV-PCSK9-injected mice, compared to less than 5% in AAV-Luciferase-injected controls (Fig. S9c). We also note a corresponding increase in total cholesterol and LDL levels in serum, which was consistent with induction of hypercholesterolemia as expected in mice injected with AAV-PCSK9 (Fig. S9d). Analysis of the liver also showed that AAV-PCSK9-injected mice had significantly lower fat deposits (Fig. S9e). Compared to controls, we observed an over 10-fold increase in macrophage infiltration, as indicated by CD45+ CD68+ cells in diseased aorta tissue (Fig. S9f). While the numbers of monocyte/macrophages in the healthier tissue were minuscule, YAP and TAZ expression in monocyte/macrophages isolated from healthy controls were not different from AAV-PCSK9-treated mice (Fig. S9f). Increased macrophage presence is also apparent from dense CD68 staining in sections of the diseased aorta and increased expression of YAP and TAZ (Fig. S10a–d). Higher vascular cell adhesion molecule 1 (VCAM1) and intercellular adhesion molecule 1 (ICAM1) expression alongside lower α-SMA in the diseased tissue are indicative of increased inflammation (Fig. S10b, e and f). There was an increased expression of oxLDL uptake receptors CD36 and LOX1 in the foam cells residing in the plaque (Fig. S10g, h). The aorta sections in the AAV-PCSK9 mice also show an increased frequency of YAP-BRD4 interactions based on proximity ligation assay (PLA) compared to AAV-Luc controls (Fig. S10i). The success of the AAV-PCSK9 model is also apparent in the mice that were fed HFD for 6 mo, where we see a significant reduction in patency of the aorta when on HFD (Fig. S9g, h).

### 3.5 BETi acts as an atheroprotective agent

**Fig. 4.**
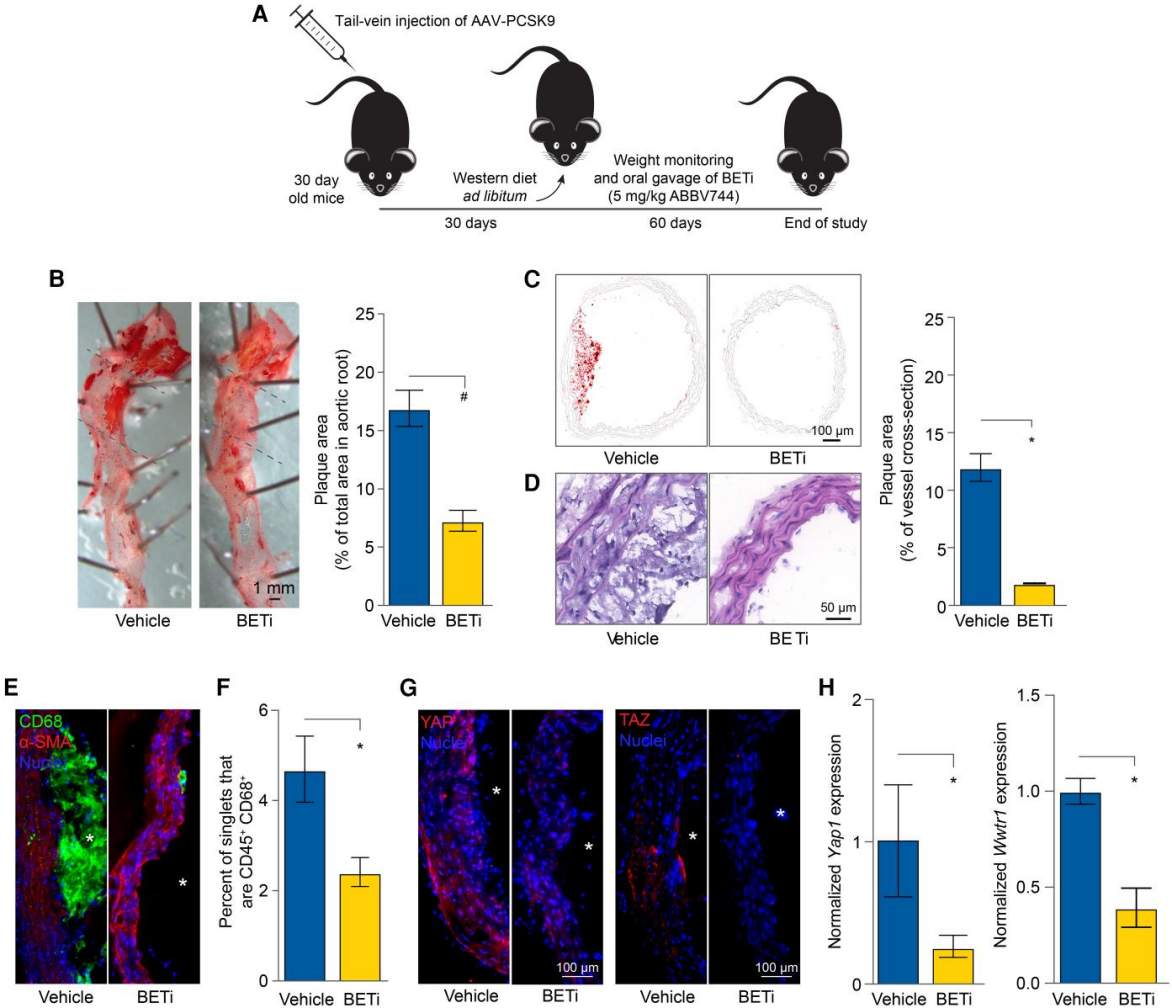
Atherogenesis is curbed in mice treated with BETi. (A) Schematic of animal study with AAV-PCSK9-injected mice, treated with vehicle and BETi, and fed HFD for 60 d. (B) *En face* Oil Red O-stained aorta (left) and corresponding plaque quantification (right) in study animals. (C) Aorta cross-sections stained with Oil Red O (left), and their corresponding quantification (right) in study animals. (D) H&E-stained aorta sections from study animals. (E) CD68 and α-SMA stained aorta sections of the study animals. (F) Percentage of macrophages recovered from the aorta of the study animals, as determined by flow cytometry. (G) YAP and TAZ staining in aorta sections of the study animals. (H) *Yap1* and *Wwtr1* expression in aorta samples from study animals, measured by qRT-PCR. Data in bar plots has been represented as mean ± SEM. # denotes *P* < 0.05 in 2-tailed unpaired Mann−Whitney *U* test, * denotes *P* < 0.05 in unpaired *t*-test. Asterisks in immunohistology images indicate the aortic lumen.

To test the effectiveness of BETi in the prevention of atherogenesis, we injected mice with AAV-PCSK9 and fed them HFD in tandem with oral dosing of the BET inhibitor ABBV-744 at 5 mg/kg *q.d.* for 2 mo. After 60 d, the animals were sacrificed, and their aorta were analyzed (Fig. 4A). BETi treatment caused a 2-fold reduction in plaque coverage at the proximal aortic arch (Fig. 4B). We also observed reduced plaque maturity in BETi-treated mice. Aorta cross-sections of these animals showed the effectiveness of BETi in curbing plaque formation (Fig. 4C). H&E staining also showed extensive plaque deposits in vehicle-control diseased mice, which were largely absent in BETi-treated mice (Fig. 4D). These heightened plaque levels were also associated with increased CD68 immunostaining (Fig. 4E) as well as increased markers associated with atherosclerosis, including VCAM1 and ICAM1 (Fig. S11a and b), and lipid uptake receptors CD36 and LOX1 (Fig. S11c and d), within aortic sections for vehicle-treated mice in comparison to BETi-treated mice. Flow cytometry analysis confirmed that a lower proportion of CD45^+^ CD68^+^ cells were isolated from the aortas of BETi-treated animals (Fig. 4F). In addition, YAP (*Yap1*) and TAZ (*Wwtr1*) expressions were curtailed in the aortas of BETi-treated animals, as quantified by immunofluorescence (Fig. 4G) and qRT-PCR (Fig. 4H).

### 3.6 BETi is atheroprotective even with YAP/TAZ overexpression in macrophages

**Fig. 5.**
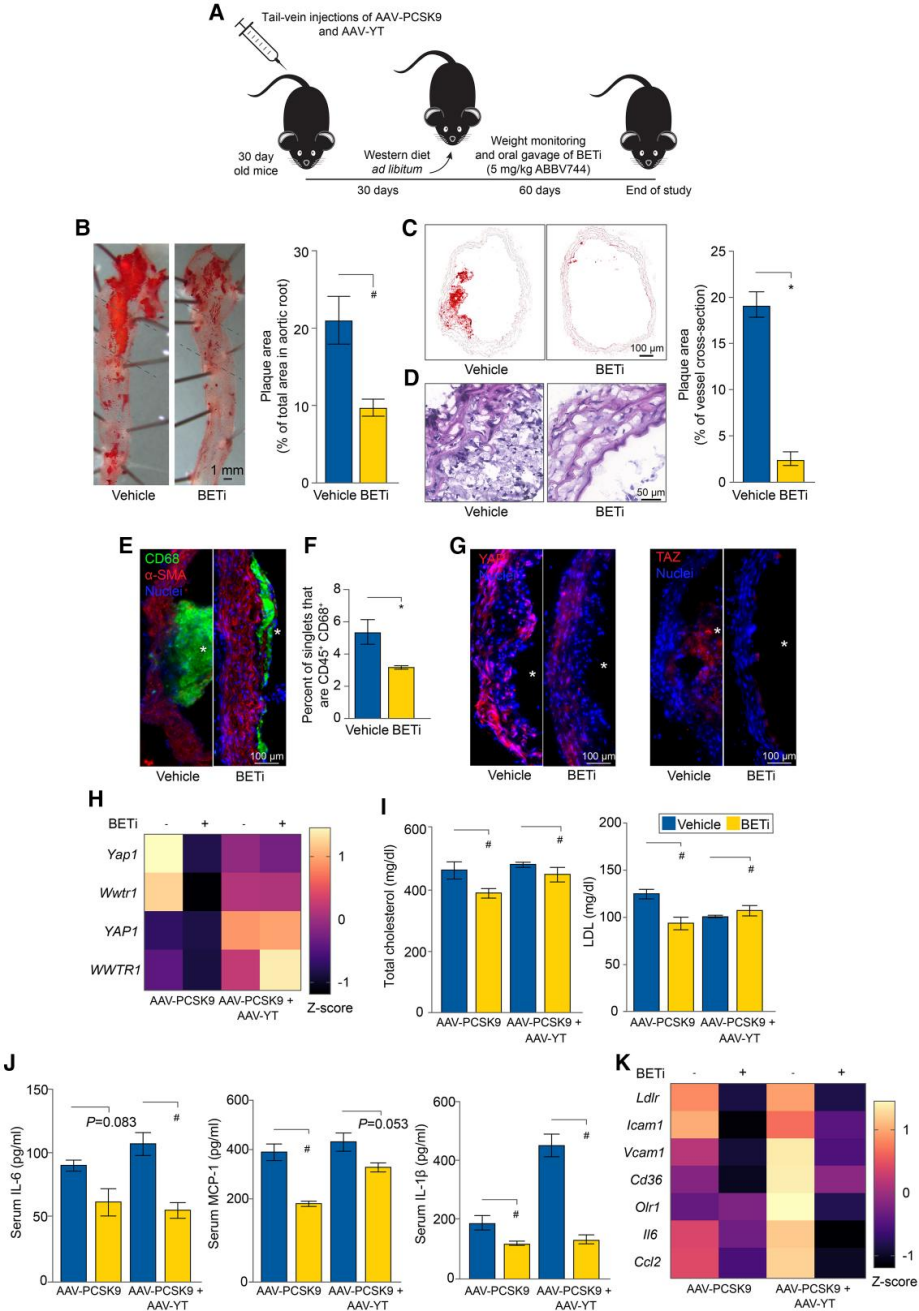
BETi-treated AAV-PCSK9 + AAV-YT animals display reduced inflammatory marker expression. (A) Schematic of animal study with mice co-injected with AAV-PCSK9 and AAV-YT, treated with vehicle and BETi, and fed HFD for 60 d. (B) *En face* Oil Red O-stained aorta and corresponding plaque quantification in AAV-PCSK9 + AAV-YT co-injected study animals. (C) Aorta cross-sections stained with Oil Red O, and their corresponding quantification in study animals. (D) H&E-stained aorta sections from study animals. (E) CD68 and α-SMA stained aorta sections of the study animals. (F) Percentage of macrophages recovered from the aorta of the study animals, as determined by flow cytometry. (G) YAP and TAZ expression in aorta sections with BETi treatment in AAV-PCSK9 + AAV-YT-injected mice. (H) Human and mouse *YAP1*/*Yap1* and *WWTR1*/*Wwtr1* gene expression in aorta tissue of test animals detected by qRT-PCR. (I) Serum levels of total cholesterol and LDL measured in the study animals. (J) Serum levels of IL-6, MCP-1, and IL-1β measured in the study animals. (K) qRT-PCR measures of gene expression of inflammatory markers (*Il6*, *Ccl2*), atherosclerosis markers (*Ldlr*, *Icam1*, *Vcam1*), and macrophage oxLDL uptake receptors (*Cd36*, *Olr1*) in the study animals. Data in bar plots has been represented as mean ± SEM. # denotes *P* < 0.05 in 2-tailed unpaired Mann−Whitney *U* test. * denotes *P* < 0.05 in unpaired *t*-test. Asterisks in immunohistology images indicate the aortic lumen.

We next studied the potential role of YAP/TAZ-BRD4 in oxLDL uptake and atherosclerosis within our established animal model. To test whether inhibition of BRD4 with BETi would be effective even in conditions of aberrantly high YAP/TAZ expression and activity (as is often noted in atherosclerosis pathogenesis^13^), we co-injected AAV-PCSK9 mice with injections of AAV designed to drive expression of human YAP^5SA^ and TAZ^S89A^ specifically in myeloid cells, under control of the Lyz2 promoter (henceforth AAV-YT) (Fig. S12a). Similar to the previously described AAV-PCSK9 mice, these mice were also fed HFD and administered BETi for 60 d, after which the animals were sacrificed and their aorta analyzed (Fig. S12b). No significant differences in weight were observed between the groups (Fig. S12c). We confirmed the effectiveness of AAV-YT injections by examining the YAP and TAZ expression in CD45^+^ CD68^+^ cells in the blood of injected animals (Fig. S13a). This was confirmed via qRT-PCR analysis of aorta tissue from AAV-YT-injected mice (Fig. 5H). BETi inhibition caused a significant reduction (>50%) in aortic plaque coverage and maturity even in our AAV-PCSK9 + AAV-YT mice, as visualized by *en face* and cross-sectional Oil Red O staining, as well as in H&E (Fig. 5A–D). The percent of the reduced cross-sectional area was notable, decreasing from 20% to less than 5% with BETi treatment.

To further probe the effects of BETi on macrophage inflammatory responses and lipid levels, we examined the expression of macrophage markers, YAP/TAZ, inflammatory markers, and lipid and inflammatory cytokines in the serum. CD68 staining in the aorta sections (Fig. 5E) and the proportion of CD45+ CD68+ cells isolated from the aortas (Fig. 5F) were reduced with BETi treatment. YAP and TAZ expression were curtailed in aortas from BETi-treated animals, as quantified by immunofluorescence and qRT-PCR (Fig. 5G, H). Interestingly, total cholesterol and LDL levels in the serum of animals were marginally yet significantly reduced with BETi treatment for both AAV-PCSK9 and AAV-PCSK-9 + AAV-YT groups (Fig. 5I). This suggests that BETi treatment may also have a systemic effect on cholesterol homeostasis in hypercholesterolemic mice. Furthermore, serum levels of inflammatory cytokines, including IL-6, IL-1β, and MCP-1, were significantly reduced with BETi treatment in AAV-PCSK9 and AAV-PCSK-9 + AAV-YT groups (Fig. 5J). Subdued levels of MCP-1 in BETi-treated animals may partly explain the reduced macrophage infiltration observed in aorta tissue. We also observed reduced expression (by qRT-PCR) of markers demarcating foam cell formation (*Ldlr*), endothelial inflammation (*Icam1*, *Vcam1*), macrophage inflammation (*Il6*, *Ccl2*), and oxLDL uptake receptors (*Cd36*, *Olr1*) in the BETi-treated mice (Fig. 5K). Finally, we find that the expression of VCAM1, ICAM1, CD36, and LOX1 evaluated by immunohistochemistry were all significantly reduced in aorta sections of BETi-treated animals (Fig. S13b–e).

### 3.7 YAP/TAZ enrichment at target gene loci is curtailed by BRD4 inhibition

**Fig. 6.**
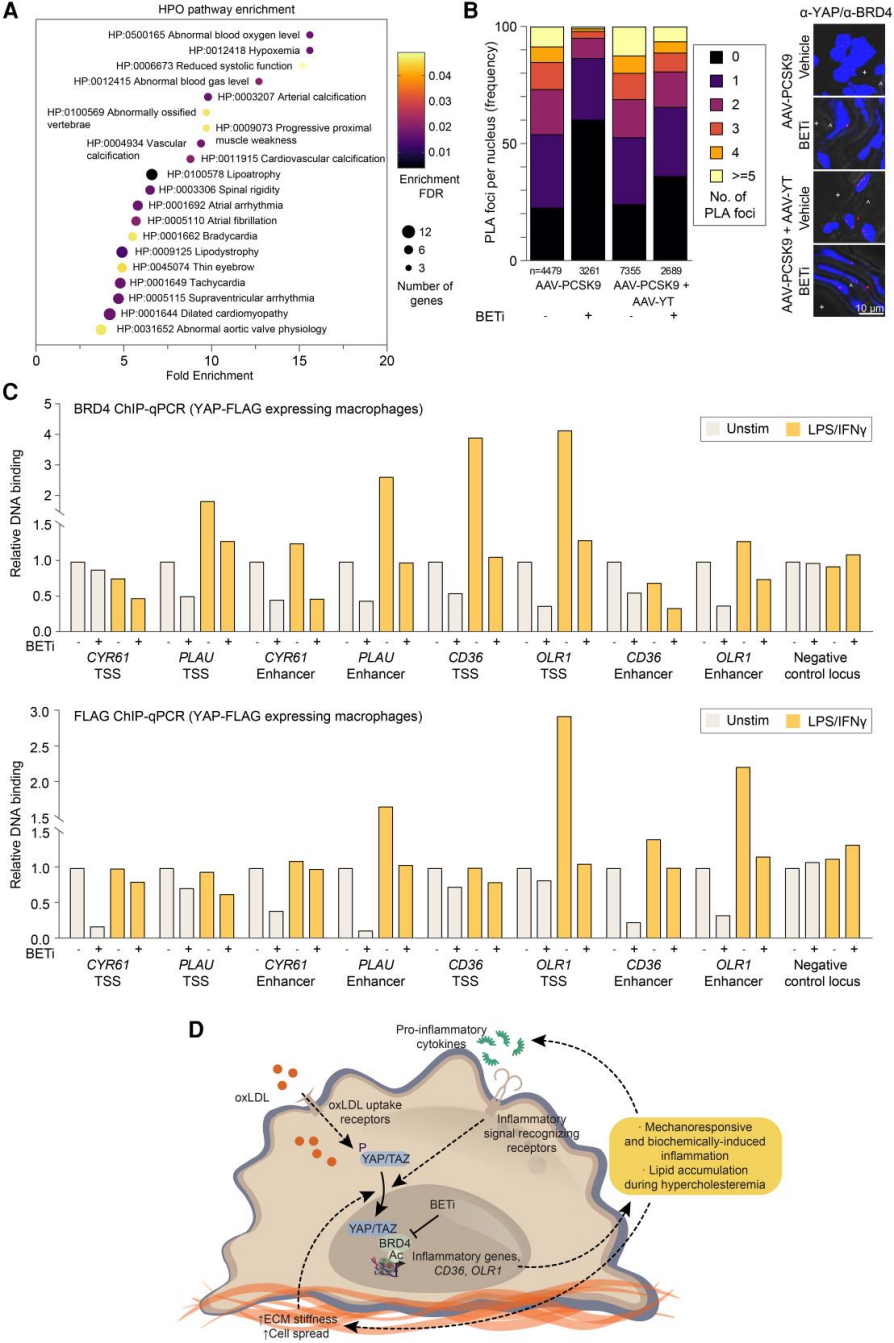
YAP/TAZ–BRD4 interactions drive atherogenesis (A) top 20 human phenotype ontology (HPO)-enriched terms for the common YAP^5SA^ and TAZ^S89A^ upregulated genes. (B) Proximity-ligation assay (PLA) quantification and representative images in aorta sections from AAV-PCSK9, and AAV-PCSK9 + AAV-YT-injected mice, treated with vehicle and BETi, and fed HFD for 60 d. Images focus on the region near the arterial wall and atherosclerotic plaque interface. Symbols: + marks the luminal edge and ^ indicates the arterial wall. (C) Chromatin immunoprecipitation (ChIP) – qPCR for BRD4 and YAP enrichment (FLAG-tagged YAP) binding on promoters and enhancers of YAP/TAZ target genes with BETi treatment. DNA enrichment was calculated as a fraction of input and presented as a fold vs binding in unstimulated cells. Data is from one of two experiments, both producing similar results. (D) Schematic summarizing the roles of macrophage YAP/TAZ and BRD4 in atherosclerosis and inflammation. Data in bar plots has been represented as mean ± SEM.

To more broadly explore the extent to which BRD4 was essential for YAP/TAZ-mediated atherosclerotic disease progression, we first performed a Disease Ontology analysis of the genes commonly upregulated in YAP^5SA^ and TAZ^S89A^ expressing macrophages (identified in Fig. 1B). The top 20 associated terms consisted of several cardiovascular and cholesterol homeostasis disorders (Fig. 6A). Interestingly, atherosclerotic tissue from mice treated with BETi exhibited fewer YAP-BRD4 interactions (PLA foci) compared to vehicle control animals (Fig. 6B), although this effect was mitigated in AAV-YT conditions. Given observations that (i) YAP/TAZ activity governed oxLDL uptake in vitro in a BRD4 dependent manner, (ii) BETi-treated hypercholesteremic mice had lower plaque deposition in their aortas, and (iii) atherosclerotic disease presents with elevated YAP/TAZ expression and activation within macrophages (and endothelial cells),^13,44–46^ we wished to explore the interdependence of YAP/TAZ/BRD4 interactions in the context of localization to their chromatin targets. Accordingly, we performed chromatin immunoprecipitation targeting nuclear YAP (FLAG), TAZ (FLAG), and BRD4, followed by qPCR (ChIP-qPCR) at the promoters and enhancers of 2 canonical YAP/TAZ targets, *CYR61* and *PLAU*, and 2 oxLDL uptake receptors, *CD36* and *OLR1,* which we show are regulated by YAP/TAZ in macrophages. Notably, we observed that BRD4 inhibition causes reduced YAP, TAZ, and BRD4 enrichment at these target gene loci (Fig. 6C, Figure S13f, g). Together, these data suggest that BRD4 is critical for YAP/TAZ-dependent expression of genes, including those that play a significant role in lipid uptake, and that BETi causes disruption of YAP and BRD4 localization to several shared chromatin targets.

## 4. Discussion

In this study, we demonstrated that YAP and TAZ interact with BRD4 and that the inhibition of BRD4 using BETi reduces inflammation and lipid uptake, which are exacerbated by YAP/TAZ activation. Given the inability of YAP/TAZ to bind to the chromatin directly, BRD4 may act as a gateway to other inflammatory factors, such as NF-κB or p300.^49,50^ We found that YAP/TAZ−BRD4 interactions, previously implicated in transcriptional addiction in cancer cells,^9^ drive inflammatory responses in macrophages. Further, there is a significant overlap in the inflammatory genes affected by the knockdown of YAP, TAZ, and BRD4. In agreement, we find that the expression of constitutively active forms of YAP and TAZ is sufficient to elicit a partial inflammatory phenotype in macrophages. This phenomenon is curtailed by BETi treatment. We also reveal that *CD36* and *OLR1* (LOX1) are among the macrophage YAP/TAZ targets relevant to the inflammatory disease of atherosclerosis.

Atherogenesis is concurrent with increased YAP and TAZ expression and activity in macrophages and endothelial cells are associated with plaque formation.^13^ Macrophages play a vital role in atherogenesis by contributing to foam cells that form the bulk of the plaque lesion. Foam cells are formed when oxLDL accumulates in macrophages and causes them to be dysfunctional. Since CD36 and LOX1 are important oxLDL uptake receptors that play vital roles in atherosclerosis, we wished to explore BETi as potential atheroprotective agents that can reduce foam cell formation and inflammation and curb disease progression. Other BRD4 inhibitors have been studied before for their anti-atherogenic properties. Intraperitoneal injections of JQ1 (50 mg/kg *q.d.*) in the *Ldlr^−/−^* mouse atherosclerosis model that was fed HFD for 10 wk showed a significant reduction in plaque coverage (2% to less than 0.5%).^5^ The study posited that NF-κB activity was hampered in inflammatory super-enhancers with JQ1 administration, reducing inflammation and atherosclerosis. YAP/TAZ may be involved in these super-enhancers to orchestrate inflammation. Another study demonstrated that oral doses of the BD2-specific BRD4 inhibitor RVX-208 (150 mg/kg *b.i.d.*) for 12 wk in an *ApoE^−/−^* atherosclerosis model led to a 39% decrease in plaque coverage (10% to 6%).^6^ The study showed a significant 2-fold decrease in serum LDL levels as the probable cause of reduced atherosclerosis with BRD4 inhibition. Our study used a different BD2-specific BRD4 inhibitor, ABBV-744 (BETi), in an AAV-PCSK9-induced atherosclerosis model. Recent findings suggest that the 2 bromodomains of the BRD4 protein may not have equivalent functions, with bromodomain 2 (BD2) being more relevant in immuno-inflammatory conditions.^51^ Thus, we chose to prioritize the study of a BRD4 inhibitor targeted to BD2 specifically. While we observed a significant reduction in inflammatory cytokines and gene expression, we noted only marginal decreases (10% to 15%) in total cholesterol and LDL levels in BETi-treated mice. Our study animals treated with BETi showed significant decreases in plaque coverage, macrophage infiltration/foam cell presence, YAP/TAZ expression, oxLDL uptake receptor expression, and various indicators of local and systemic inflammation. BETi was proficient in atheroprotection even in the mice that received the AAV-YT injections that cause elevated YAP/TAZ expression in monocyte/macrophage cells, hinting at its effectiveness in curbing YAP/TAZ activation-associated inflammation and oxLDL uptake/foam cell formation. This work motivates future work aimed at more directly linking the YAP/TAZ-BRD4 axis to the atherogenic effects of YAP/TAZ and BRD4 and the atheroprotective effects of BETi.

In addition to exerting anti-inflammatory effects on macrophages, systemic BETi administration may affect additional cell types including endothelial cells and vascular smooth muscle cells (VSMCs) involved in atherosclerosis. YAP and TAZ are expressed by endothelial cells and are responsive to hemodynamic forces. In atheroprone areas, disturbed blood flow patterns activate YAP/TAZ, which subsequently promotes endothelial cell proliferation and contributes to the progression of atherosclerosis.^45,46,52^ In vascular smooth muscle cells (VSMCs), a deficiency of tissue factor pathway inhibitor-1 (TFPI-1) drives atherogenesis by increasing proliferation and migration. This pro-atherogenic effect is mediated by the phosphorylation of angiomotin (AMOT), which releases YAP from cytoplasmic sequestration and subsequently activates it.^53^ Future experiments involving myeloid-targeted BET inhibition may be needed to elucidate the YAP/TAZ-BRD4 axis further in macrophages and understand its specific impact on atherogenesis.

A previous study in cancer cells has shown that YAP/TAZ recruits BRD4 to the enhancers of its target genes to enhance transcriptional addiction.^9^ Inflammation and loss of elastic laminae in atherosclerotic vessels result in up to 100% thicker and 50% stiffer vascular walls.^54^ The known roles of YAP/TAZ in macrophage mechanosensation^17^ and fibrotic gene transcription^55^ also suggest the presence of a cycle of perpetual inflammation ie driven by stiffening ECM, YAP/TAZ activation, inflammatory transcription, and oxLDL uptake ie caused by inflammatory polarization of macrophages and transformation into foam cells (Fig. 6D). BRD4 inhibition using BETi serves as a promising intervention that affects this cycle at various points, providing redundancy through its potent anti-inflammatory effects.

## Supplementary Material

qiag017_Supplementary_Data

## Data Availability

Data will be made available upon request.
