# Supplementary material for: BET inhibition curbs macrophage inflammation, lipid accumulation, and atherogenesis by disrupting the YAP/TAZ-BRD4 axis
Source: J Leukoc Biol. 2026 Apr 29;118(7):qiag017. doi: 10.1093/jleuko/qiag017 (PMC13412306; doi:10.1093/jleuko/qiag017)
Supplement: qiag017_Supplementary_Data [file qiag017_supplementary_data.pdf]

Supplementary Material

BET inhibition curbs macrophage inflammation, lipid accumulation, and atherogenesis by disrupting the YAP/TAZ-BRD4 axis

Praveen Krishna Veerasubramanian[1,2], Vijaykumar S. Meli[1,2], Hamza Atcha[1,2], Wenqi Wang[3], Timothy L. Downing[1,2,4,5,*], Wendy F. Liu[1,2,6,7,8,*]

[1]Department of Biomedical Engineering

[2]UCI Edwards Lifesciences Foundation Cardiovascular Innovation and Research Center (CIRC)

[3]Department of Developmental and Cell Biology

[4]NSF-Simons Center for Multiscale Cell Fate Research

[5]Department of Microbiology and Molecular Genetics

[6]Chemical and Biomolecular Engineering

[7]Department of Molecular Biology and Biochemistry

[8]Institute for Immunology

*Corresponding Authors

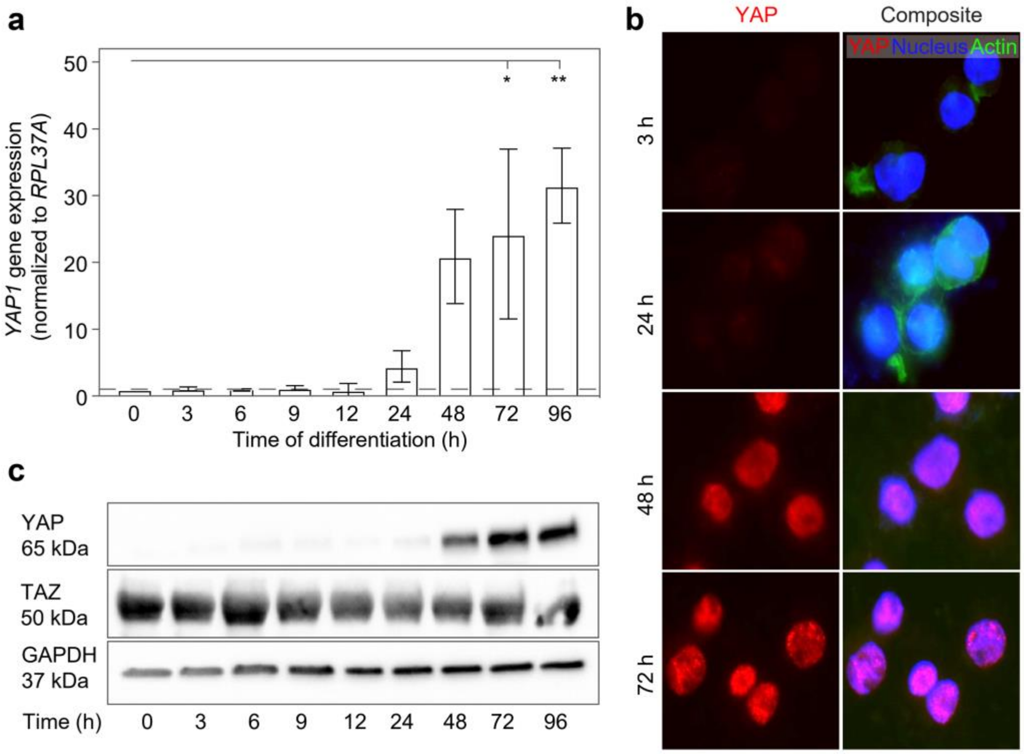

Supplementary Fig. 1. YAP and TAZ are expressed in macrophages differentiated from THP-1 cells. (**a**) *YAP1* gene expression measured by qRT-PCR in THP-1 cells differentiated to macrophages with phorbol 12-myristate 13-acetate (PMA) for 4 d, normalized to *RPL37A*. (**b**) YAP immunofluorescence stained THP-1 derived macrophages. (**c**) Western blot of time course of YAP and TAZ expression levels in THP-1 derived macrophages. Data in bar plots has been represented as mean ± SEM. * denotes $P<0.05$ in unpaired Kruskal-Wallis test with Dunn post-hoc multiple comparisons.

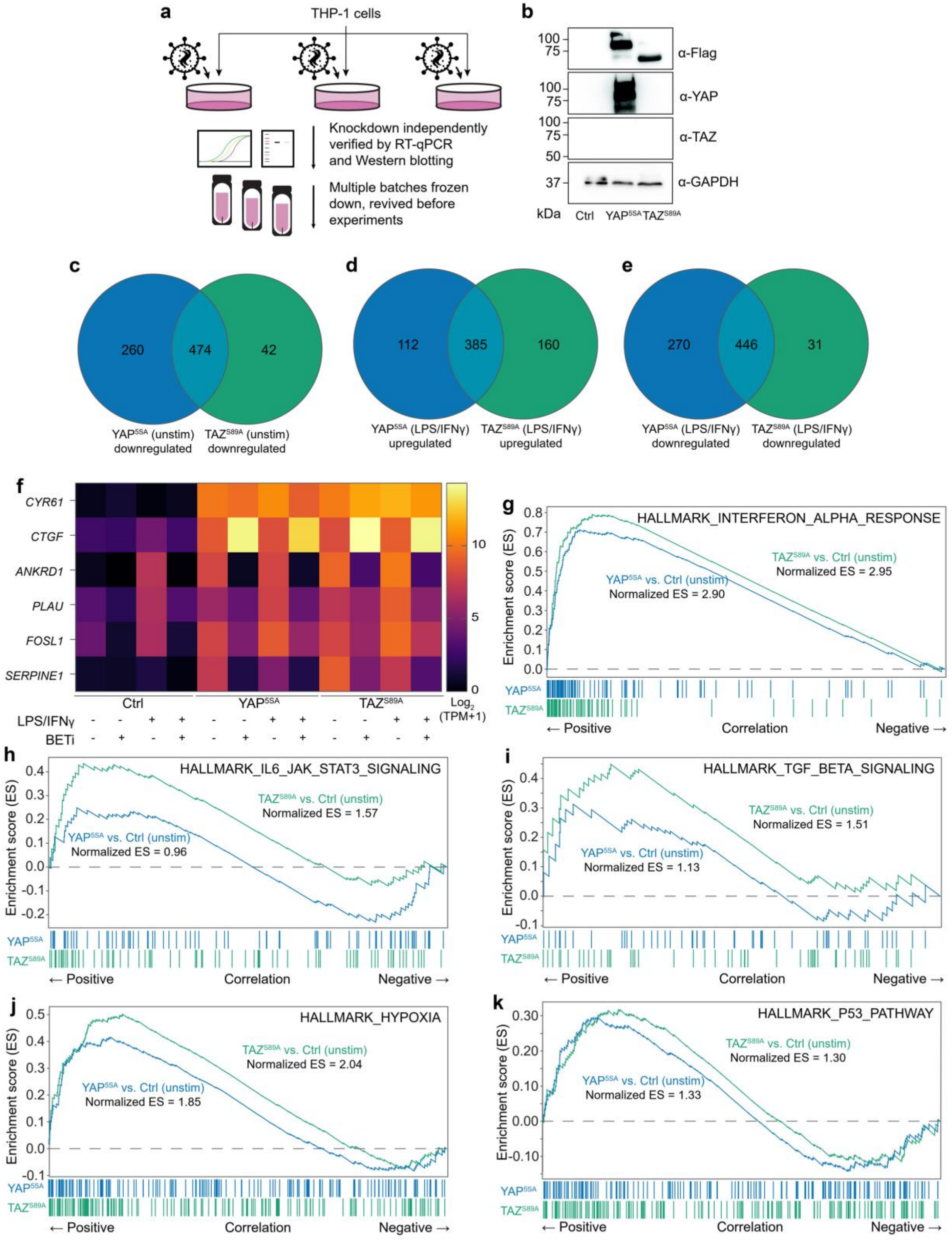

a
THP-1 cells
Knockdown independently verified by RT-qPCR and Western blotting
Multiple batches frozen down, revived before experiments
b
α-Flag
α-YAP
α-TAZ
α-GAPDH
kDa
Ctrl
YAP5SA
TAZS89A
c
260
474
42
YAP5SA (unstim) downregulated
TAZS89A (unstim) downregulated
d
112
385
160
YAP5SA (LPS/IFNγ) upregulated
TAZS89A (LPS/IFNγ) upregulated
e
270
446
31
YAP5SA (LPS/IFNγ) downregulated
TAZS89A (LPS/IFNγ) downregulated
f
CYR61
CTGF
ANKRD1
PLAU
FOSL1
SERPINE1
Ctrl
YAP5SA
TAZS89A
LPS/IFNγ
BETi
Log2 (TPM+1)
g
HALLMARK_INTERFERON_ALPHA_RESPONSE
TAZS89A vs. Ctrl (unstim)
Normalized ES = 2.95
YAP5SA vs. Ctrl (unstim)
Normalized ES = 2.90
Enrichment score (ES)
← Positive
Correlation
Negative →
h
HALLMARK_IL6_JAK_STAT3_SIGNALING
TAZS89A vs. Ctrl (unstim)
Normalized ES = 1.57
YAP5SA vs. Ctrl (unstim)
Normalized ES = 0.96
i
HALLMARK_TGF_BETA_SIGNALING
TAZS89A vs. Ctrl (unstim)
Normalized ES = 1.51
YAP5SA vs. Ctrl (unstim)
Normalized ES = 1.13
j
HALLMARK_HYPOXIA
TAZS89A vs. Ctrl (unstim)
Normalized ES = 2.04
YAP5SA vs. Ctrl (unstim)
Normalized ES = 1.85
k
HALLMARK_P53_PATHWAY
TAZS89A vs. Ctrl (unstim)
Normalized ES = 1.30
YAP5SA vs. Ctrl (unstim)
Normalized ES = 1.33

Supplementary Fig. 2. YAP and TAZ overexpression induces inflammation in macrophages. (**a**) Schematic for lentiviral mediated overexpression cells derived from THP-1. (**b**) Western blots validating the overexpression of YAP, TAZ and the presence of Flag tag in the engineered YAP^{5SA} and TAZ^{S89A} cells, in comparison to an empty vector control (Ctrl). (**c**) Significant overlap exists between the genes downregulated in unstimulated cells with YAP^{5SA} and TAZ^{S89A} expression in comparison to the control unstimulated cells. (**d, e**) Significant overlap exists between the genes upregulated and downregulated in LPS/IFNγ-stimulated cells with YAP^{5SA} and TAZ^{S89A} expression in comparison to the control LPS/IFNγ-stimulated cells. (**l**) Canonical YAP/TAZ target genes are upregulated in YAP^{5SA} and TAZ^{S89A} cells but suppressed when THP-1 cells are treated with BETi. (**g-k**) Increased enrichment for hallmark interferon alpha response, IL6/JAK/STAT3, TGF-β, hypoxia response, and P53 pathway genes identified by GSEA in YAP^{5SA} and TAZ^{S89A} expressing cells (ES: Enrichment score). Violin plots show quartiles and median. # denotes *P*<0.05 in paired Friedman test with Dunn's post-hoc for multiple comparisons.

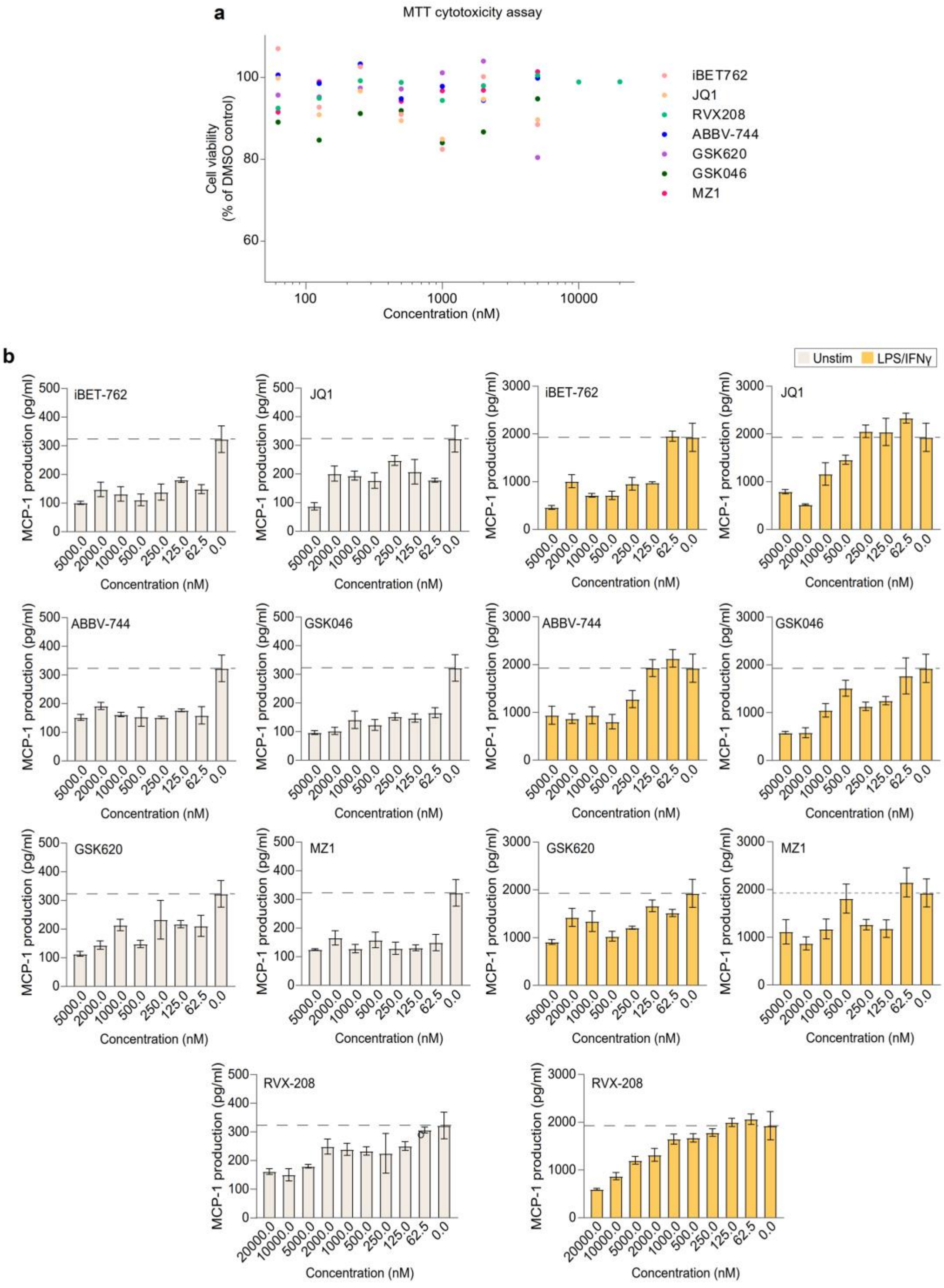

Supplementary Fig. 3. BETi using various pharmacological agents are widely anti-inflammatory without affecting macrophage viability. (**a**) MTT cytotoxicity assay measuring viability after 24 h exposure to various BETi treatments. (**b**) MCP-1 protein secretion measured using ELISA in THP-1 cells pre-treated with BETi drugs for 24 h prior to 24 h of stimulation with LPS/IFNγ if applicable. Data in bar plots has been represented as mean ± SEM.

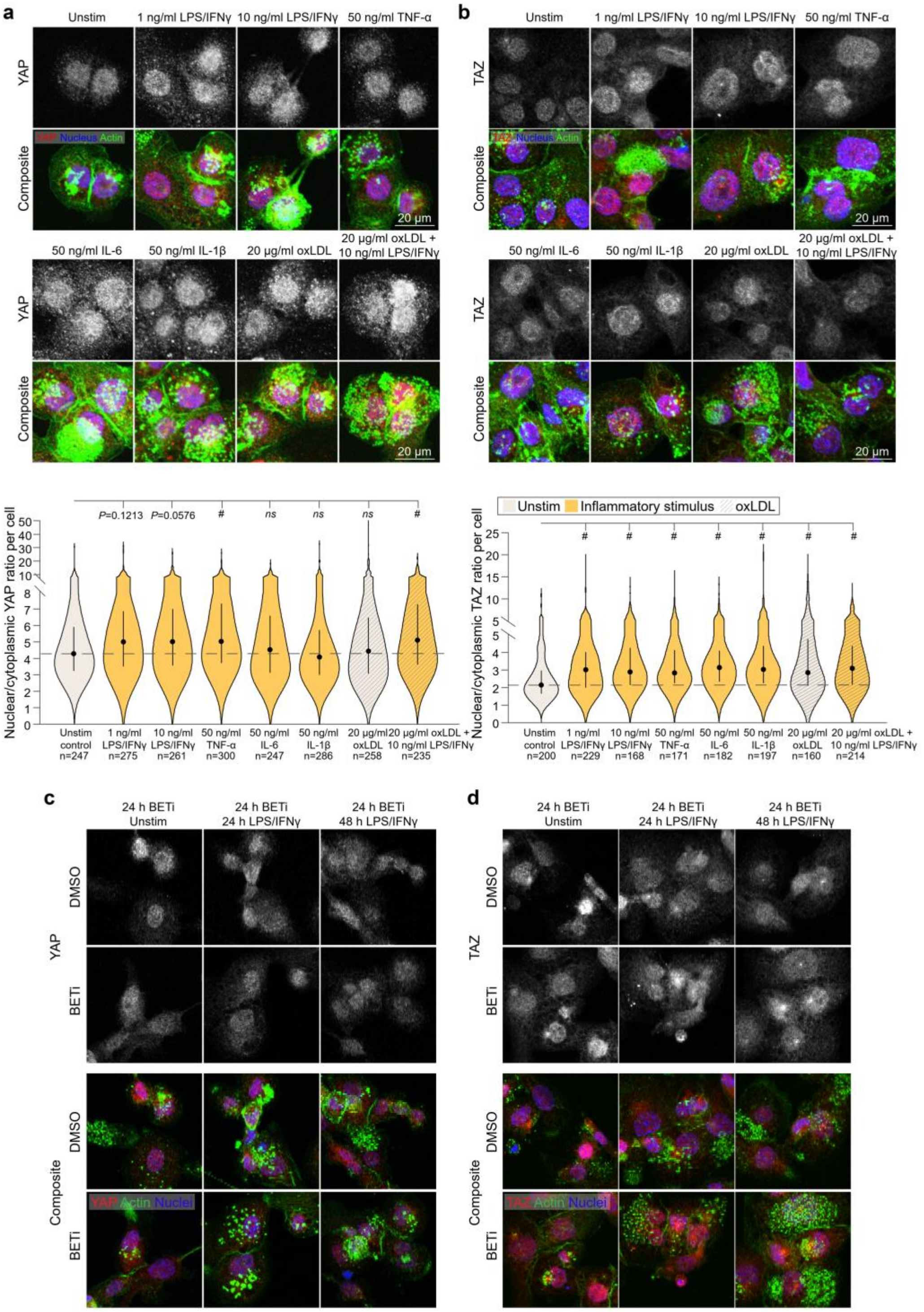

Supplementary Fig. 4. YAP and TAZ expression and nuclear translocation are both modulated by polarizing stimuli, but not by BETi. (**a**) Representative images of YAP immunostained THP-1 cells, quantification of nuclear-to-cytoplasmic ratio after 6 h of stimulatory cytokine treatments. (**b**) Representative images of TAZ immunostained THP-1 cells, quantification of nuclear-to-cytoplasmic ratio after 6 h of stimulatory cytokine treatments. (**c**) Representative images of YAP and TAZ immunostained cells after 24 h of BETi treatment, followed by 0 h, 24 h or 48 h of LPS/IFNγ stimulation. Violin plots show quartiles and median. # denotes *P*<0.05 in unpaired Kruskal-Wallis test with Dunn post-hoc multiple comparisons.

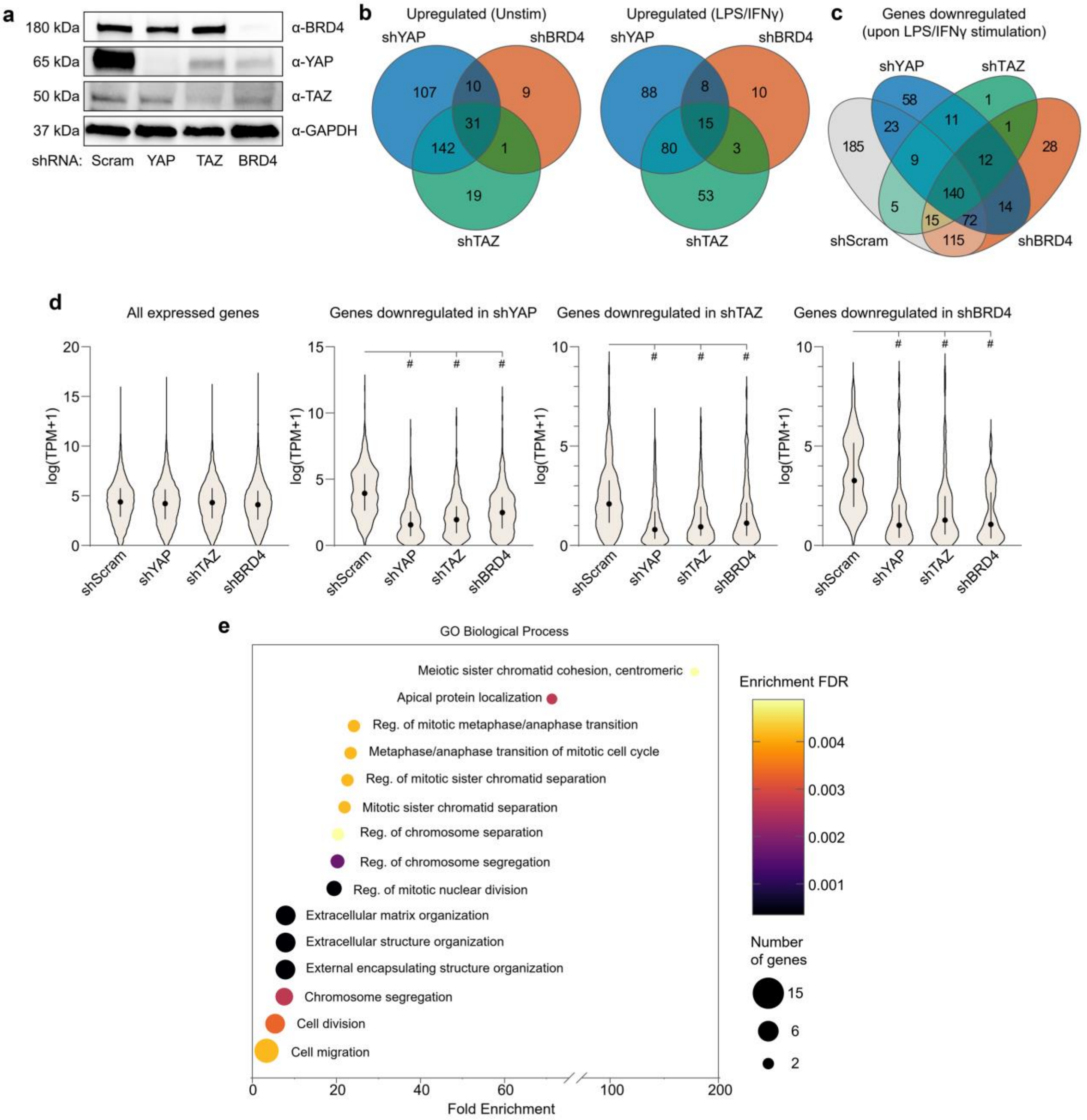

Supplementary Fig. 5. The loss of YAP, TAZ, and BRD4 have common anti-inflammatory effects in macrophages. (**a**) Western blots validating the knockdown of YAP, TAZ, and BRD4 using shRNA treatment in THP-1 cells, compared to shScram control. (**b**) Significant overlap exists between the genes that are downregulated in shYAP, shTAZ and shBRD4 expressing cells in comparison to shScram controls, in both unstimulated and LPS/IFNγ-stimulated cells. (**c**) Genes downregulated with knockdown conditions upon LPS/IFNγ treatment. (**d**) Gene expression patterns of all expressed genes, shYAP downregulated genes, shTAZ downregulated genes, and shBRD4 downregulated in shRNA-treated unstimulated THP-1 cells. (**e**) Top 15 biological process gene ontology (GO) terms enriched in the subset of genes downregulated commonly by shYAP, shTAZ, shBRD4 treatments compared to shScram control when unstimulated. Violin plots show quartiles and median. # denotes *P*<0.05 in paired Friedman test with Dunn's post-hoc for multiple comparisons.

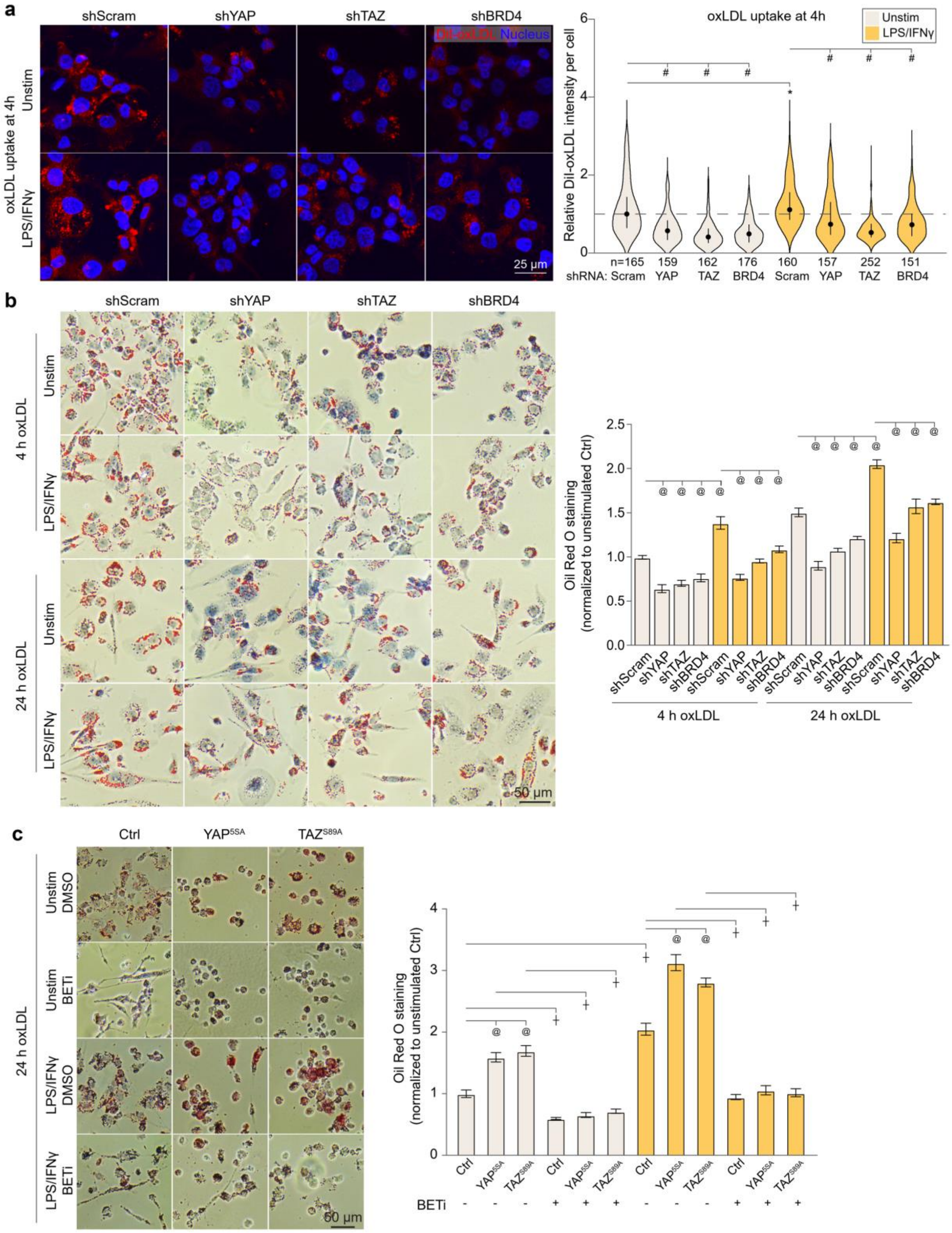
a
shScram
shYAP
shTAZ
shBRD4
DiI-oxLDL Nucleus
oxLDL uptake at 4h
Unstim
LPS/IFNγ
25 µm
oxLDL uptake at 4h
Unstim
LPS/IFNγ
Relative DiI-oxLDL intensity per cell
n=165 159 162 176 160 157 252 151
shRNA: Scram YAP TAZ BRD4 Scram YAP TAZ BRD4
b
shScram
shYAP
shTAZ
shBRD4
4 h oxLDL
24 h oxLDL
Unstim
LPS/IFNγ
50 µm
Oil Red O staining
(normalized to unstimulated Ctrl)
shScram shYAP shTAZ shBRD4 shScram shYAP shTAZ shBRD4 shScram shYAP shTAZ shBRD4 shScram shYAP shTAZ shBRD4
4 h oxLDL
24 h oxLDL
c
Ctrl
YAP5SA
TAZS89A
24 h oxLDL
Unstim DMSO
Unstim BETi
LPS/IFNγ DMSO
LPS/IFNγ BETi
50 µm
Oil Red O staining
(normalized to unstimulated Ctrl)
Ctrl YAP5SA TAZS89A Ctrl YAP5SA TAZS89A Ctrl YAP5SA TAZS89A Ctrl YAP5SA TAZS89A
BETi - - - + + + - - - + + +

Supplementary Fig. 6. Macrophage oxLDL uptake is modulated by YAP, TAZ, and BRD4 expression. (**a**) Representative images and quantification of DiI-oxldl uptake at 4 h in knockdown cells under unstimulated and LPS/IFNγ-stimulated conditions. (**b**) Representative images and quantification of Oil Red O stain in macrophages at 24 h post oxLDL exposure in knockdown cells under unstimulated and LPS/IFNγ-stimulated conditions. (**c**) Representative images and quantification of Oil Red O stain in macrophages at 24 h post oxLDL exposure in YAP^{5SA} and TAZ^{S89A} expressing cells under unstimulated and LPS/IFNγ-stimulated conditions. Data in bar plots has been represented as mean ± SEM. Violin plots show quartiles and median. * denotes *P*<0.05 in two-tailed unpaired Mann-Whitney U test. # denotes *P*<0.05 in unpaired Kruskal-Wallis test with Dunn post-hoc multiple comparisons. @ denotes *P*<0.05 in ordinary one-way ANOVA with Tukey's multiple comparisons test. ┼ denotes *P*<0.05 in unpaired t-test.

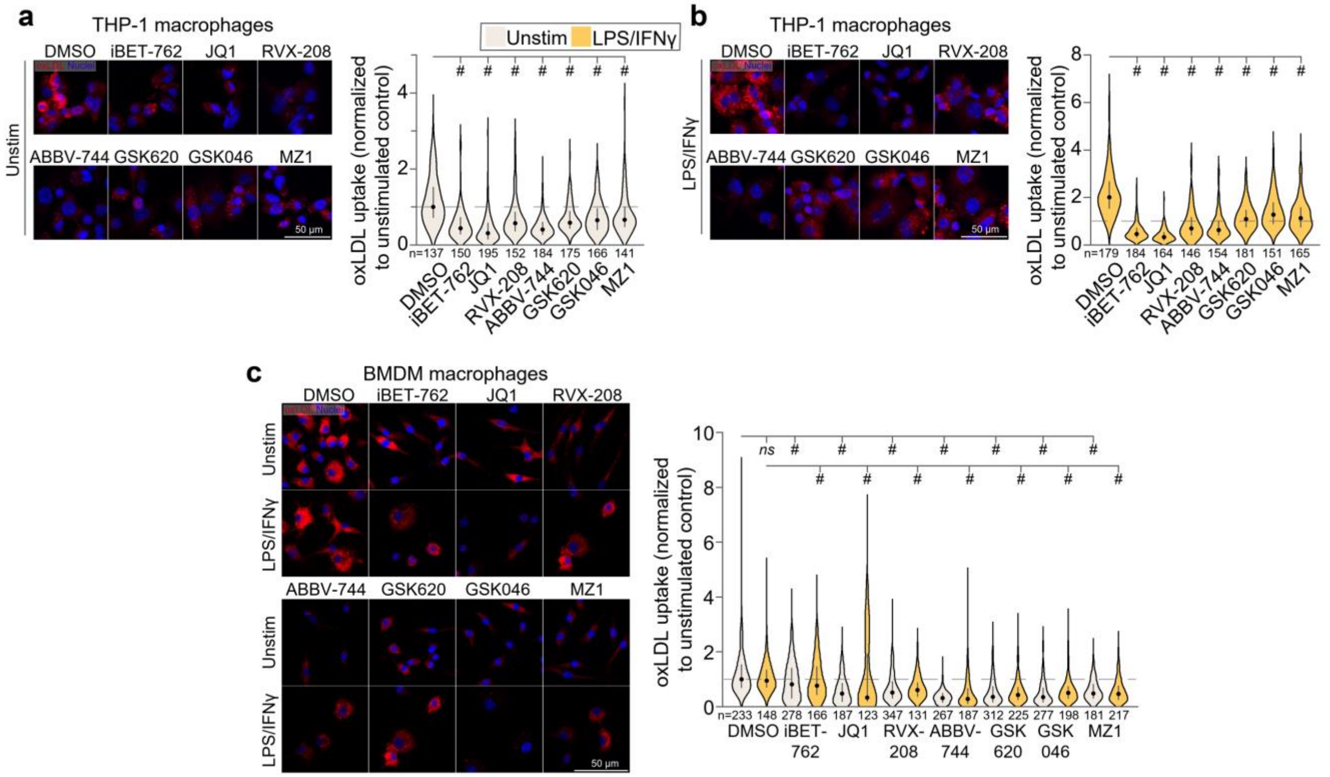

Supplementary Fig. 7. BETi causes reduced uptake of oxLDL in mouse and human macrophages. (**a**) Representative images and quantification of DiI-oxLDL uptake at 24 h, post 24 h of BETi treatment in THP-1 macrophages, without inflammatory stimulation. (**b**) Representative images and quantification of DiI-oxLDL uptake at 24 h, post 24 h of BETi treatment in THP-1 macrophages, in LPS/IFNγ-stimulated cells. (**c**) Representative images and quantification of DiI-oxLDL uptake at 24 h, post 24 h of BETi treatment in mouse bone marrow-derived macrophages (BMDM), in unstimulated and LPS/IFNγ-stimulated cells. Data in bar plots has been represented as mean ± SEM. Violin plots show quartiles and median. # denotes *P*<0.05 in unpaired Kruskal-Wallis test with Dunn post-hoc multiple comparisons. * denotes *P*<0.05 in unpaired t-test.

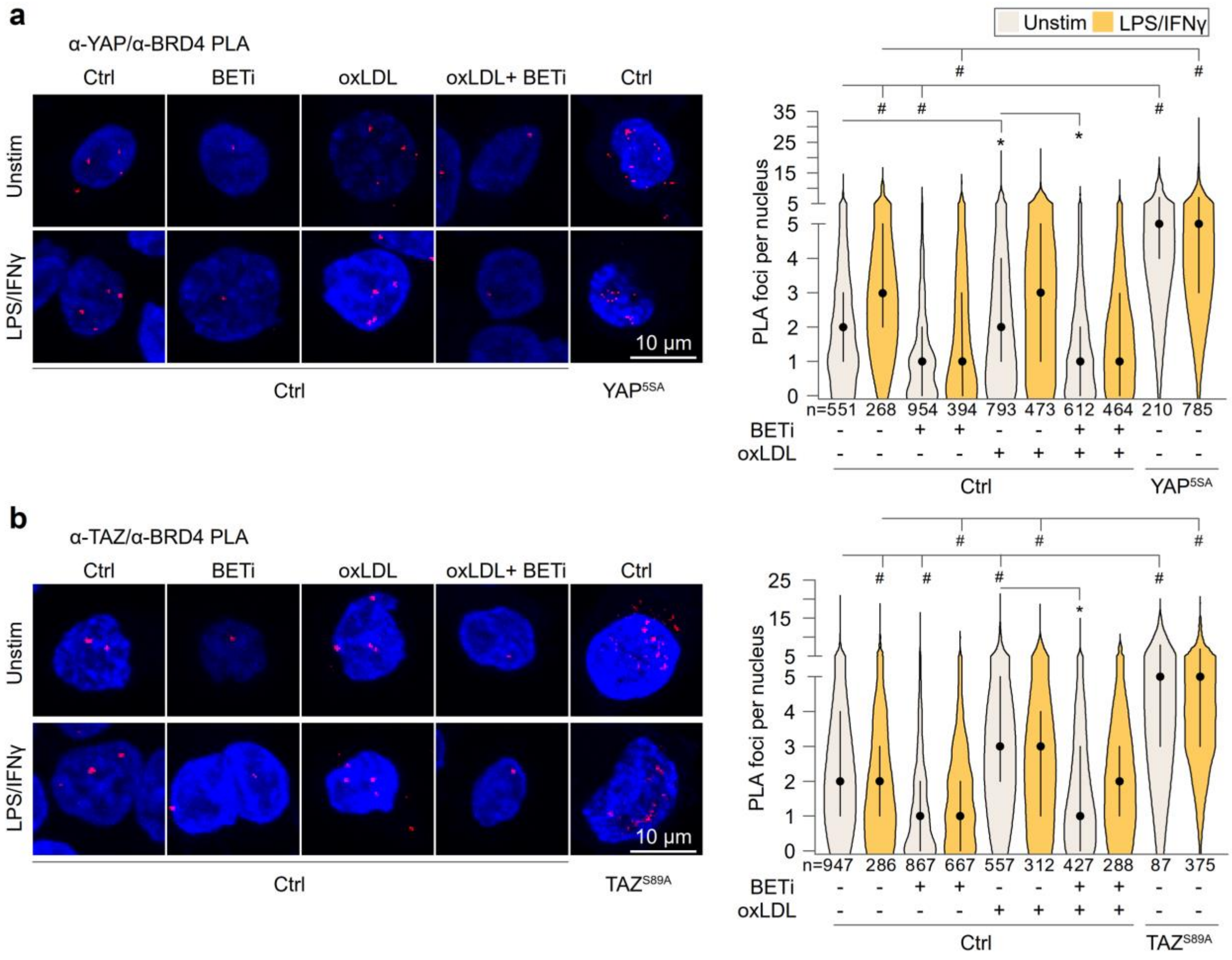

Supplementary Fig. 8. oxLDL treatment causes increased interactions between YAP-BRD4 and TAZ-BRD4 in THP-1 cells. (**a**) Representative images of proximity ligation (PLA), and quantification of nuclear PLA foci between YAP and BRD4 in THP-1 cells under different treatments. (**b**) Representative images of proximity ligation (PLA), and quantification of nuclear PLA foci between TAZ and BRD4 in THP-1 cells under different treatments. Violin plots show quartiles and median. * denotes *P*<0.05 in two-tailed unpaired Mann-Whitney U test. # denotes *P*<0.05 in unpaired Kruskal-Wallis test with Dunn post-hoc multiple comparisons.

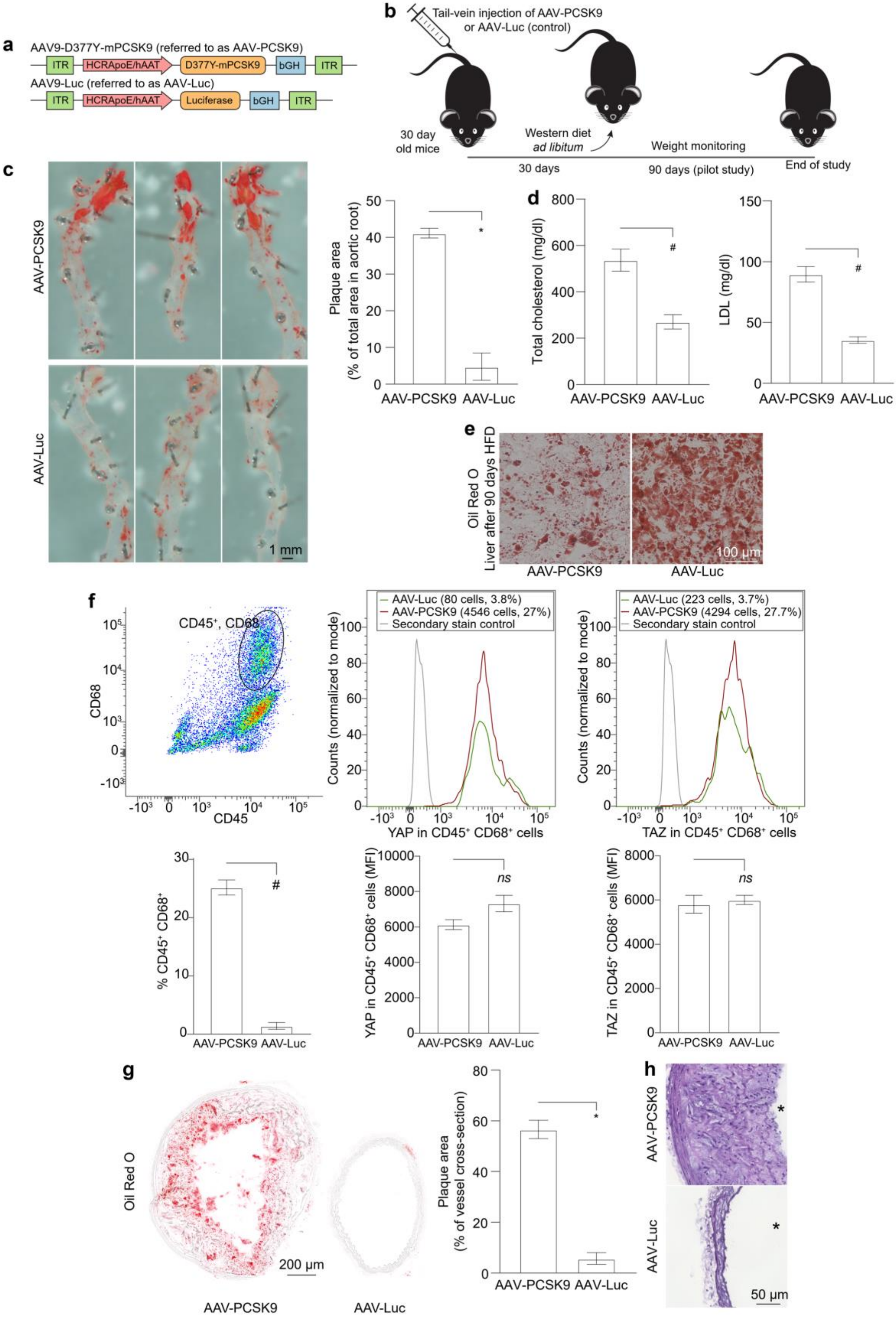
a
AAV9-D377Y-mPCSK9 (referred to as AAV-PCSK9)
ITR
HCRApoE/hAAT
D377Y-mPCSK9
bGH
ITR
AAV9-Luc (referred to as AAV-Luc)
Luciferase
b
Tail-vein injection of AAV-PCSK9
or AAV-Luc (control)
30 day
old mice
Western diet
ad libitum
Weight monitoring
30 days
90 days (pilot study)
End of study
c
AAV-PCSK9
AAV-Luc
1 mm
Plaque area
(% of total area in aortic root)
d
Total cholesterol (mg/dl)
LDL (mg/dl)
e
Oil Red O
Liver after 90 days HFD
100 µm
f
CD45+, CD68+
CD68
CD45
AAV-Luc (80 cells, 3.8%)
AAV-PCSK9 (4546 cells, 27%)
Secondary stain control
Counts (normalized to mode)
YAP in CD45+ CD68+ cells
AAV-Luc (223 cells, 3.7%)
AAV-PCSK9 (4294 cells, 27.7%)
TAZ in CD45+ CD68+ cells
% CD45+ CD68+
YAP in CD45+ CD68+ cells (MFI)
TAZ in CD45+ CD68+ cells (MFI)
ns
g
Oil Red O
200 µm
Plaque area
(% of vessel cross-section)
h
50 µm

Supplementary Fig. 9. AAV-mediated PCSK9 GOF model of atherogenesis. (**a**) AAV-PCSK9 constructs used in this paper. (**b**) Schematic of the study timeline for the initial pilot study used to establish the model, and the study that assessed the efficacy of BETi in curbing atherogenesis. (**c**) *En face* Oil Red O-stained aorta and corresponding plaque quantification in AAV-PCSK9-injected mice, and AAV-Luc control mice, and fed HFD for 90 days as part of the pilot study. (**d**) Total cholesterol and LDL levels in serum of AAV-PCSK9-injected mice, and AAV-Luc control mice, and fed HFD for 90 days. (**e**) Representative Oil Red O staining images of liver sections from AAV-PCSK9-injected mice, and AAV-Luc control mice, and fed HFD for 90 days, showing extensive deposition of fat in mice liver in AAV-Luc controls, but not in the AAV-PCSK9 livers. (**f**) Gating strategy, quantification of CD45+ CD68+ cells (macrophages/monocytes) isolated from digested aorta tissue in the pilot study animals, YAP and TAZ staining in CD45+ CD68+ cells determined by flow cytometry of cells from digested aorta of pilot study animals. (**g**) Aorta cross-sections stained with Oil Red O, and their corresponding quantification in the pilot study animals, at 6 months on HFD. (**h**) H&E-stained aorta sections from the pilot study animals, at 6 months on HFD. Data in bar plots has been represented as mean ± SEM. # denotes $P<0.05$ in two-tailed unpaired Mann-Whitney U test. * denotes $P<0.05$ in unpaired t-test. Asterisks in immunohistology images indicate aortic lumen.

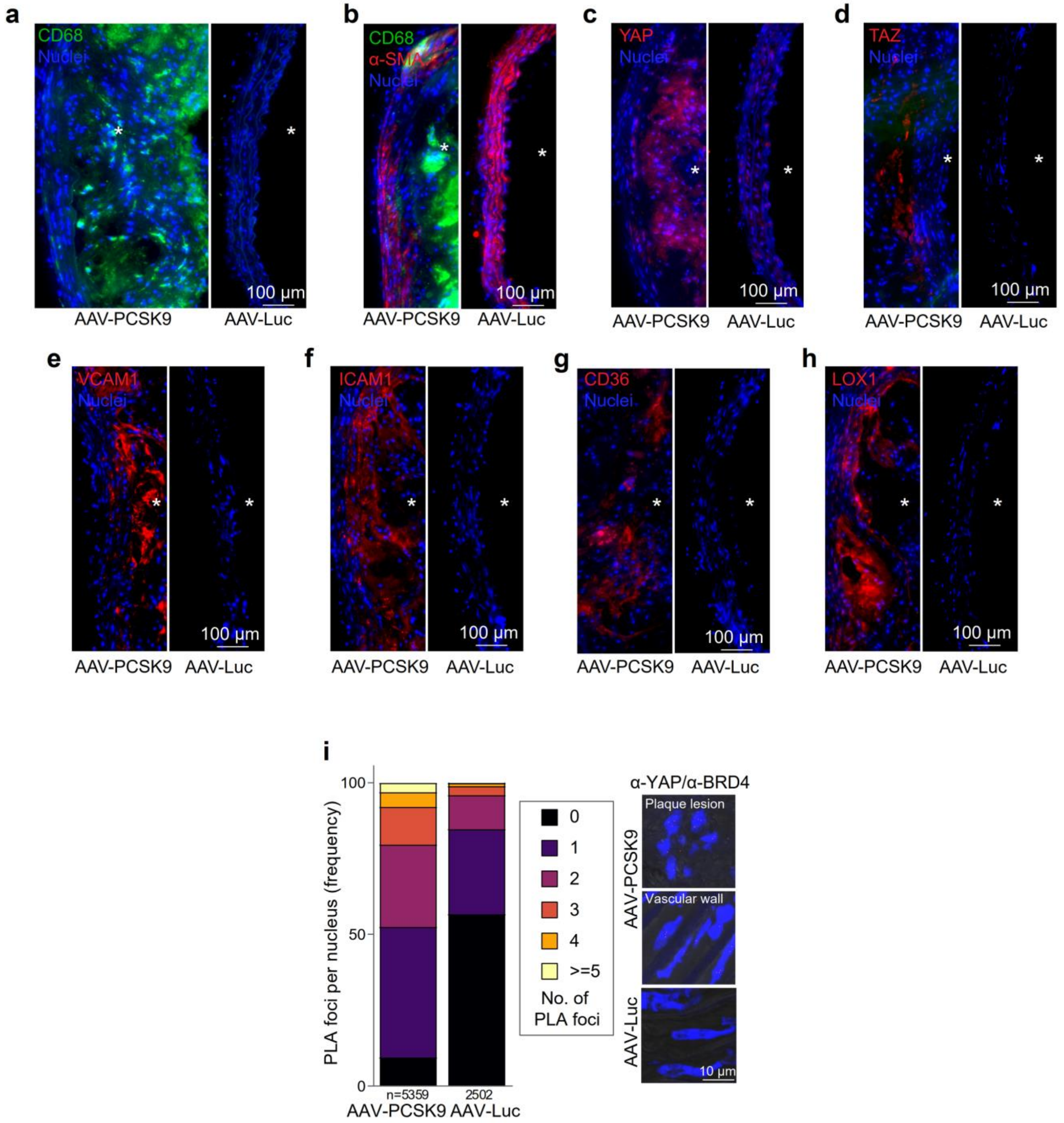

Supplementary Fig. 10. Immunofluorescence staining of atherosclerosis markers in pilot study animals. (**a**) CD68, (**b**) α-SMA, (**c**) YAP, (**d**) TAZ, (**e**) VCAM1, (**f**) ICAM1, (**g**) CD36, and (**h**) LOX1 immunofluorescence staining in the aorta from the pilot study animals (AAV-PCSK9-injected mice, and AAV-Luc control mice, fed HFD for 90 days). (**i**) Representative images of proximity ligation (PLA), and quantification of nuclear PLA foci between YAP and BRD4 in aorta tissue slices from pilot study animals. Asterisks in immunohistology images indicate aortic lumen.

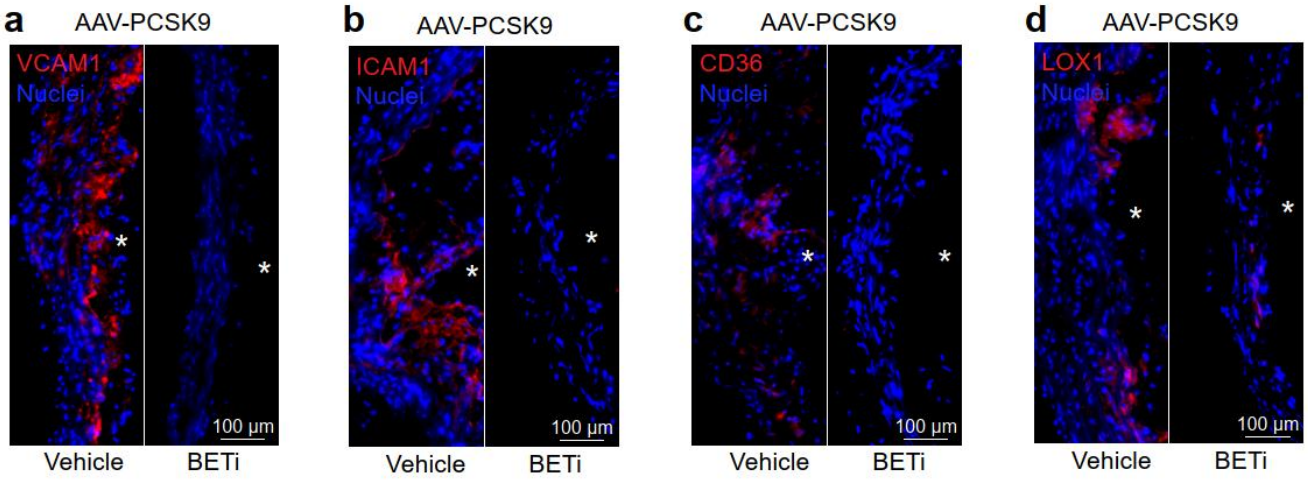

Supplementary Fig. 11. BETi results in decreased expression of atherosclerosis markers study animals. (**a**) VCAM1, (**b**) ICAM1, (**c**) CD36, and (**d**) LOX1 immunofluorescence staining in the aorta from the BETi study animals (AAV-PCSK9-injected mice fed HFD for 60 days with concurrent BETi oral treatments). Asterisks in immunohistology images indicate aortic lumen.

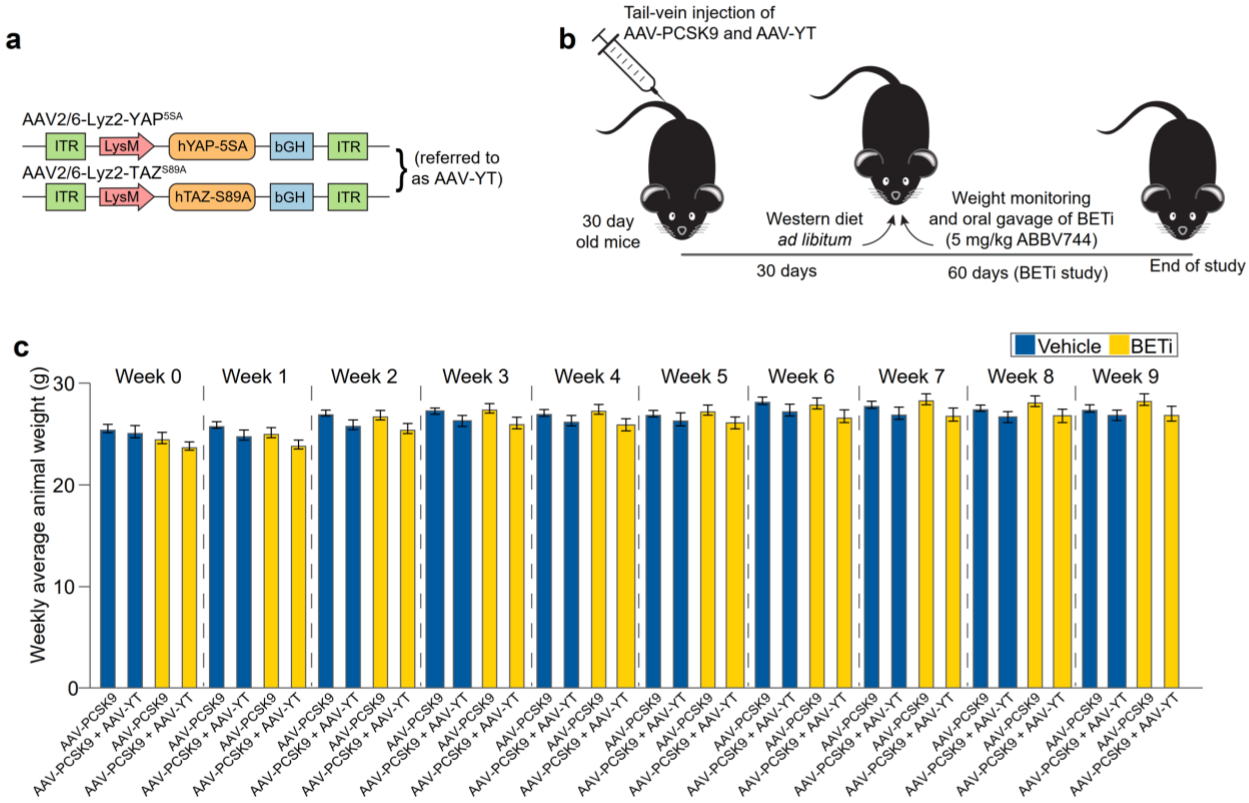

Supplementary Fig. 12. Schematic of AAV-PCSK9 + AAV-YT study (**a**) AAV constructs used to induce YAP and TAZ overexpression in monocyte/macrophages (**b**) Schematic explaining experimental setup of the study (**c**) Animal weights from the BETi treatment study shows no discernable changes in body weight with BETi treatment. Data in bar plots has been represented as mean ± SEM.

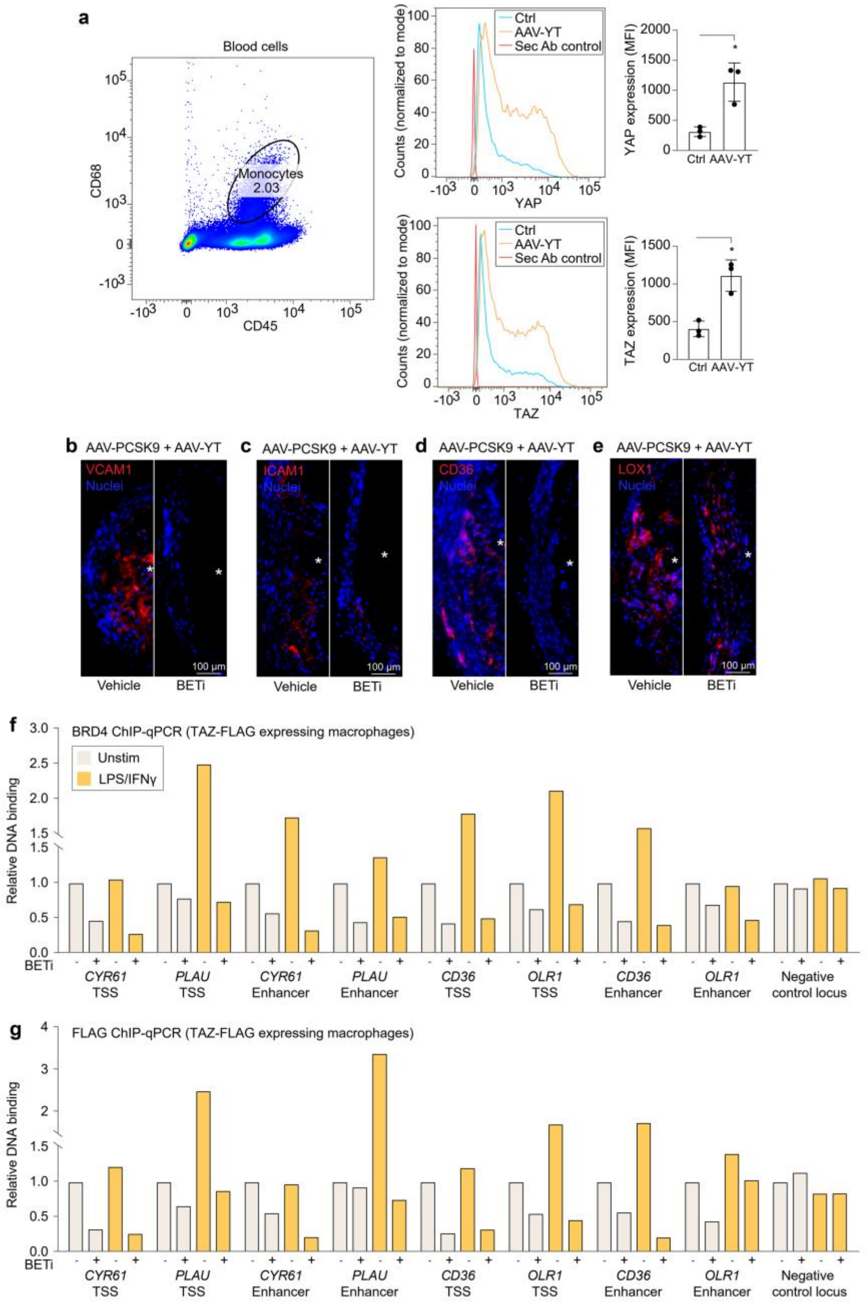

Supplementary Fig. 13. YAP and TAZ expression are higher in monocyte/macrophages in mice injected with AAV-YT. BETi results in decreased expression of atherosclerosis markers study animals. (**a**) Gating strategy for flow cytometry on blood cells from mice, targeting CD45+ CD68+ cells (monocytes), histogram and quantification of YAP and TAZ expression in CD45+ CD68+ cells in AAV-YT mice and Ctrl mice shows successful increase in YAP and TAZ expression in the monocyte population. (**b**) VCAM1, (**c**) ICAM1, (**d**) CD36, and (**e**) LOX1 immunofluorescence staining in the aorta from the BETi study animals (AAV-PCSK9 + AAV-YT injected mice, fed HFD for 60 days with concurrent BETi oral treatments). Asterisks in immunohistology images indicate aortic lumen. (**f-g**) Chromatin immunoprecipitation (ChIP) – qPCR showing reduced BRD4 and TAZ (FLAG-tagged TAZ) binding on promoters and enhancers of YAP/TAZ target genes with BETi treatment. DNA enrichment was calculated as a fraction of input, and presented as a fold versus binding in unstimulated cells. Data is from one of two experiments, both producing similar results. Data in bar plots has been represented as mean ± SEM. * denotes P<0.05 in unpaired t-test.

Extended Data Tables

Table 1. List of shRNA sequences used to generate the knockdown cell lines in this study

shRNA	Sequence
**shYAP-1**	5'-AAGCTTTGAGTTCTGACATCC-3'
**shYAP-2**	5'-CTGGTCAGAGATACTTCTTAA-3'
**shTAZ-1**	5′-CAGACATGAGATCCATCACTA-3′
**shTAZ-2**	5′-TACTTCCTCAATCACATAGAA-3′
**shBRD4-1**	5'-CCAACCAAAGTCAGTTCCTTC-3'
**shBRD4-2**	5'-CAGTGACAGTTCGACTGATGA-3'
**shScram**	5'-CCTAAGGTTAAGTCGCCCTCG-3'

Table 2. List of qRT-PCR primers used in this study

	Gene name	Forward	Reverse
**Human**	*YAP1*	TGTCCCAGATGAACGTCACAGC	TGGTGGCTGTTTCACTGGAGCA
	WWTR1	GAGGACTTCCTCAGCAATGTGG	CGTTTGTTCCTGGAAGACAGTCA
	CD36	CAGGTCAACCTATTGGTCAAGCC	GCCTTCTCATCACCAATGGTCC
	OLR1	GAAACCCTTGCTCGGAAGCTGA	CAGATCCAGTCTTGCGGACAAG
	TNF	AGGCGCTCCCCAAGAAGACAGG	CAGCAGGCAGAAGAGCGTGGTG
	IL6	AGACAGCCACTCACCTCTTCAG	TTCTGCCAGTGCCTCTTTGCTG
	CCL2	CCCCAGTCACCTGCTGTTAT	TGGAATCCTGAACCCACTTC
	IL1B	CCGACCACCACTACAGCAAG	GGGCAGGGAACCAGCATCTT
**Mouse**	*Yap1*	CCCGACTCCTTCTTCAAGC	CTCGAACATGCTGTGGAGTC
	Wwtr1	GTCACCAACAGTAGCTCAGATCC	GTTGCTGAGGAAGTCTTCTGGAG
	Ldlr	GAATCTACTGGTCCGACCTGTC	CTGTCCAGTAGATGTTGCGGTG
	Vcam1	GCTATGAGGATGGAAGACTCTGG	ACTTGTGCAGCCACCTGAGATC
	Icam1	AAACCAGACCCTGGAACTGCAC	GCCTGGCATTTCAGAGTCTGCT
	Il6	TAGTCCTTCCTACCCCAATTTCC	TTGGTCCTTAGCCACTCCTTC
	Ccl2	TTAAAAACCTGGATCGGAACCAA	GCATTAGCTTCAGATTTACGGGT
	Cd36	GGACATTGAGATTCTTTTCCTCTG	GCAAAGGCATTGGCTGGAAGAAC
	Olr1	GTCATCCTCTGCCTGGTGTTGT	TGCCTTCTGCTGGGCTAACATC

Table 3. List of ChIP-PCR primers used in this study

Locus type	Associated gene	Forward	Reverse
TSS	*CYR61*	CACACACAAAGGTGCAATGGAG	CCGGAGCCCGCCTTTTATAC
	PLAU	CCTCAGTCCAGACGCTGTTG	CTCCCTCCCCTGTCTTGCAG
	CD36	ATTTGGCTCAGGTGTCAGGG	ATGAGGCACAGGCTCTCAAC
	OLR1	GCTGGTCCTTCACAGTCTGG	CGTGACTGCTTCACTCTCTCA
Enhancer	*CYR61*	ACTGTTAGGGAGCGGACACT	AGAAATCGCAAGGGCACAAAC
	PLAU	GCTGGCTTCACCCTTCACAC	ATGGGGCAGACGGACTCTTC
	CD36	CTCTCTTCCGATTCCCCAGC	CCCCTGCCCCTTGAGATTTT
	OLR1	CTTTTTCAATACCATCTCCTCCGC	TCTAGAACAGACTCCGAGGGA
Control	Negative control locus	CTGCCTAGGGCACATTCCAA	CCCCATCTCCAGTACCCTGA
